# A high-resolution phenotypic screen identifies regulators of CNS axon diameter in zebrafish

**DOI:** 10.1371/journal.pbio.3003895

**Published:** 2026-07-17

**Authors:** Maria A. Eichel-Vogel, Daniel Y. H. Soong, Melody N. Sequeira, Maya L. A. Ibenfeldt, Katy L. H. Marshall-Phelps, Jenea M. Bin, David A. Lyons

**Affiliations:** 1 Institute for Neuroscience and Cardiovascular Research, University of Edinburgh, Edinburgh, United Kingdom; 2 Simons Initiative for the Developing Brain, University of Edinburgh, Edinburgh, United Kingdom; 3 MS Society Edinburgh Centre for Multiple Sclerosis Research, University of Edinburgh, Edinburgh, United Kingdom; 4 Zebrafish Imaging and Screening Facility, University of Edinburgh, Edinburgh, United Kingdom; University of Notre Dame, Center for Stem Cells and Regenerative Medicine, UNITED STATES OF AMERICA

## Abstract

The molecular mechanisms underpinning neuronal morphogenesis remain to be fully understood. A powerful step in uncovering the molecular basis of biological processes is the execution of discovery screens in model systems. Here, we employed zebrafish to identify molecules that regulate axon diameter in the central nervous system. Axon diameter can vary by >100-fold, with larger axons exhibiting faster conduction velocity. Axon diameter influences myelination, can be dynamically regulated, and is altered in disease. However, gaining insights into axon diameter has been challenging due to the small size and complexity of axons in vivo. Therefore, we developed an automated high-resolution live imaging and analysis screening platform to identify pharmacological modulators of the diameter of the large Mauthner axon in zebrafish. We screened 880 compounds and identified 33 hits. Validating this pipeline, we confirmed that compounds affecting beta-2 adrenoceptor and dopamine signaling increase axon diameter. This represents the first subcellular in vivo imaging-based screen to detect changes in axon diameter, providing entry points to study the biology of this key feature of neuronal morphology.

## Abstract 2

Axon diameter is important for action potential conduction speed, but what molecular pathways influence this neuronal property? This study reports a high-resolution imaging-based chemical screening pipeline for a zebrafish axon, and identifies regulators of axon diameter that represent candidates for mechanistic investigation.

## Introduction

Our understanding of the mechanisms that determine and regulate the complexity of cellular morphology and function in vivo remain to be fully understood. In particular, our understanding of what controls the enormous diversity and complexity of neurons in the central nervous system of vertebrates is incomplete. Major insights into the growth and connectivity of neurons have derived from phenotypic discovery screens carried out in model organisms, including Drosophila, C elegans and zebrafish, that have identified factors required for axonal pathfinding, innervation and synapse formation [1–9]. However, additional features of neuronal morphology have remained relatively intractable. For example, our understanding of what controls the axonal shape, particularly the diameter of axons, is relatively limited. This is largely due to difficulties in visualizing the diversity of and dynamic changes to axon diameter in intact systems or circuits.

Across the vertebrate nervous system, for example, the diameter of axons can vary by >100-fold, from ~0.1 µm to tens of microns, equating to ~10,000-fold difference in cross-sectional area occupied by distinct axons [10]. The ~100-fold difference in axonal diameter is accompanied by a roughly 100-fold difference in the speed of action potential conduction along axons, with larger axons conducting proportionally faster [11–13]. In addition to differences between axons, diameter can vary along individual axons, including between branches, and over time. Axon diameter can be dynamically regulated via cell-cell interactions [14], including signals from myelinating glia cells, particularly in the PNS [15–20] and neuronal activity in healthy conditions [21–28]. Further, axon diameters are altered in a range of diseases and injuries [29–36]. Our understanding of the molecular mechanisms underpinning axon diameter growth, modulation, or changes in disease remain largely unclear, with insights primarily relating to the relationship between the axonal cytoskeleton and axon size [15,37–41]. For example, positive correlations between axon diameter and neurofilament density and phosphorylation, particularly for larger myelinated axons have been observed [15,37,42]. In smaller, unmyelinated axons, microtubule organization supports axon diameter growth [10,37]. More recently, it has been reported that the neuronal actomyosin network, particularly alpha-adducin, an actin capping protein, controls the diameter growth of axonal actin rings, thereby maintaining axonal diameters and integrity [40,41] (reviewed by [43]). Most of these insights into the biology of axon diameter have been driven by the analysis of fixed tissue preparations by high-resolution imaging approaches such as electron microscopy and super-resolution imaging. However, these approaches are not yet experimentally scalable and so discovery-based screens applied to axon diameter have not yet been carried out in model systems.

Recently, we established zebrafish as a model to study axon diameter and used transgenic reporters to observe axon diameter growth in vivo over time [20,44]. Following a genetic screen for new genes required for myelinated axon formation, we identified the nuclear transport receptor ipo13 as being essential for the growth in diameter of very large axons [20]. This capacity motivated us to develop a discovery platform that aimed to specifically identify novel regulators of axon diameter.

To perform a screen for axon diameter, we reasoned that it would be critical to use an imaging platform that allows us to resolve even subtle changes to the diameter of individual axons in vivo. We also sought to automate this process to increase scalability for screening. Our previous studies have shown that the Mauthner axon is well suited to the study of axon diameter in vivo, due to it being distinctly identifiable and exhibiting robust and early axon diameter growth during the development and functional maturation of a well-characterized neural circuit. Here, we describe the development of an automated imaging and machine learning (ML)-based analysis pipeline to assess changes to Mauthner axon diameter in the zebrafish, which grows several microns in diameter over its first few days of development [20]. With this, we executed a large chemical-compound-based discovery screen and validated hit compounds that increased axon diameter. Our work highlights the potential to screen for subtle changes to cell morphology in vivo, which could also be employed to assess other poorly understood features of neuronal structure. Further, we provide the findings from our screen as a resource for the community to accelerate investigation into the biology of axon diameter.

## Results

### Automated high-resolution imaging of axon diameter in vivo using zebrafish larvae

To investigate the molecular regulation of axon diameter in the CNS, we chose the Mauthner neurons and their axons as a model. The Mauthner neurons are a pair of reticulospinal CNS neurons located in the hindbrain and critical for mediating fast escape responses that project long large-diameter axons along the length of the spinal cord to drive motor outputs [45–47].

Our previous assessments of the Mauthner axon diameter employed high-resolution confocal live imaging of fluorescent reporters, which indicated the axon grows from ~1 to ~5 µm in diameter between 2- and 5-days post-fertilization (dpf) [20,44]. Such measurements of diameter were carried out either manually or using semi-automated analyses, unsuitable to scalable measurements. Therefore, to implement methods suited to a scalable discovery screen, we aimed to automate both the imaging and analysis of Mauthner axon diameter, balancing high-resolution imaging and high-throughput.

We previously employed automated live imaging of zebrafish expressing fluorescent reporters to screen for compounds that influenced myelinating glial cell number [48]. To do so, we employed a spinning disk confocal microscope (SDCM) for image acquisition, coupled to the vertebrate automated screening technology (VAST) platform for the automated delivery of zebrafish larvae from a 96-well plate to the microscope for imaging.

**Fig 1:**
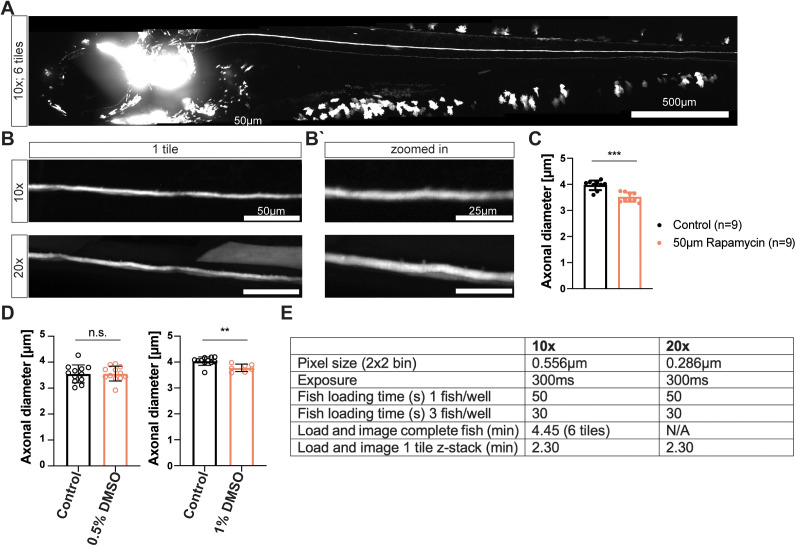
Axon diameter changes can be visualized and assessed using an automated screening system. **(A)** Maximum intensity projection of images of Tg(hspGFF62A:Gal4); Tg(UAS:GFP) zebrafish following tiled merging of 6 z-stacks. Z-stacks were acquired from lateral view with a 10×/0.5NA objective using VAST-SDCM system. Scale bar = 500 µm). **(B)** Maximum intensity projection of 1 tile z-stack at approximately somite 15–16 of Tg(hspGFF62A:Gal4); Tg(UAS:GFP) fish imaged with a 10×/0.5NA (top) or a 20×/1NA objective (bottom). Scale bars = 50 µm B22) Zoom in of B shows improved subcellular resolution at 20× compared to 10×. Scale bars = 25 µm. **(C)** Script-based semi-automatic quantifications of Mauthner axon diameter show that we can measure axon diameter changes using the 20×/1NA objective and imaging parameters in **B.** When applying the mTor inhibitor Rapamycin we detect a significant decrease in Mauthner axon diameter (*n* = 9, ***p < 0.0001). **(D)** Quantifications of Mauthner axon diameter of larvae kept in either E3 embryo medium (controls) or in 0.5% (left panel) or 1% DMSO (right panel). When applying 1% DMSO we detect a significant decrease in Mauthner axon diameter (*n* > 7, *p* = 0.0034). We detect no difference when applying 0.5% DMSO (*n* = 12, *p* = 0.94). **(E)** Imaging resolution and times for 2 × 2 binning levels using either 10× or 20× objective and imaging Tg(hspGFF62A:Gal4); Tg(UAS:GFP) reporter line at 300 ms exposure at the VAST-SDCM. The data underlying this figure can be found in S1 Data.

The VAST platform delivers zebrafish to a thin-walled glass capillary (600–750 µm bore), in which they are stabilized and automatically oriented to visualize the animal from a specific orientation. The system then hands control to the SDCM to acquire images of the animal within the glass capillary. Here, we used the transgenic reporter Tg(hspGFF62A:Gal4); Tg(UAS:GFP) [8,49] to image growth in the diameter of the Mauthner axon. Due to Mauthner axons being close to midline axis, we needed to use a long-working-distance objective to image through the tissue overlying the axons and the glass capillary. We tested both 10× and 20× objectives and acquisition protocols that imaged either the entire animal (entire length of the Mauthner axon, Fig 1A) or just one defined region (mid-way along axonal projection, Fig 1B) (see Methods and comparison of parameters shown in Fig 1E). This indicated that imaging one z-stack at 20× magnification provided the optimal parameters that balanced acquisition resolution and throughput necessary for a screen.

Next, we wanted to ensure that images acquired in this way allowed us to detect experimentally induced changes in axon diameter. We performed manipulations after the Mauthner axon has completed its outgrowth, reasoning that this would also limit identifying false positive candidates that influenced axon diameter by affecting development or earlier stages of neuronal maturation. We first assessed whether we could detect changes in axon diameter induced by manipulating the mTor pathway, which has been shown to affect axon diameter [50,51]. To do so, we applied the mTor inhibitor Rapamycin at 50 µM from 3 to 5 dpf, during active diameter growth, and were indeed able to detect a decrease in Mauthner axon diameter of around 10% upon Rapamycin treatment (with *n* = 9) (Fig 1C). It is important to note that while DMSO at 0.5% does not impact axon diameter growth, 1% DMSO significantly decreases Mauthner axon diameter compared to larvae kept in E3 embryo medium (controls) (Fig 1D). To screen ~1,000 compounds for their potential to alter axon diameter at this scale, we anticipated needing to acquire ~10,000 z-stacks from 10,000 animals (*n* ~ 10), including controls and occasional acquisition failures. This scale of analysis motivated us to develop an entirely automated image analysis pipeline.

### Creating an automated image analysis pipeline to measure axon diameter changes at subcellular resolution

**Fig 2:**
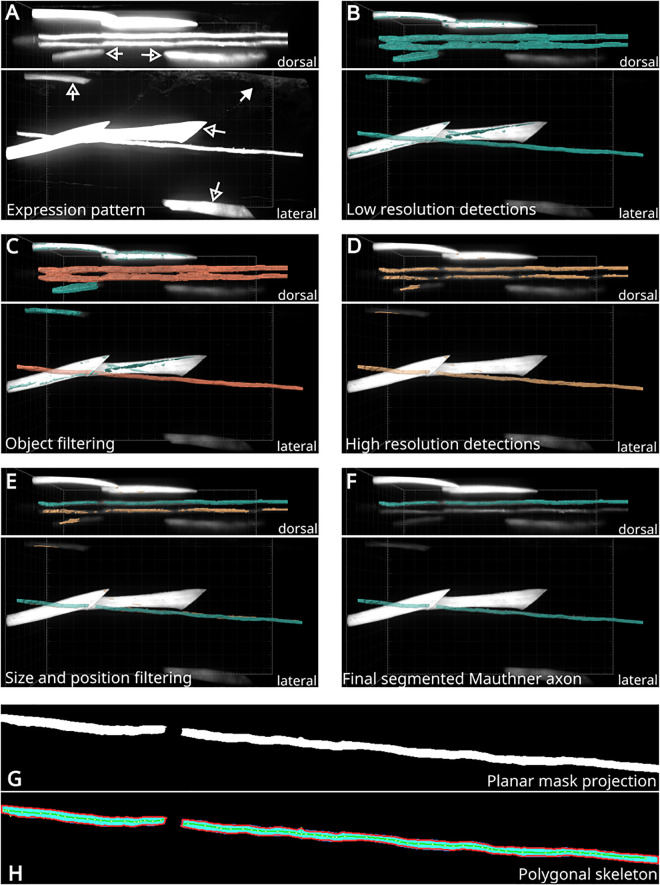
Creating an automated image analysis pipeline to measure axon diameter changes: Stepwise object detection and processing. Front and top 3D views of z-stacks rendered in Arivis Vision4D showing: **(A)** Overly contrasted grayscale look-up-table to highlight problematic skin reflection/autofluorescence (solid arrow), and ectopic muscle expression of GFP (hollow arrows). **(B)** Low-resolution ML model trained to exclude skin and muscle leaving candidate axonal objects (green). **(C)** Object filtering to exclude small objects (green), leaving target Mauthner axons (orange). **(D)** High-resolution ML model trained to tightly segment clear and reliably measure candidate axons (yellow). **(E)** Object size filtering followed by a clustering python script determines objects most likely to be the Mauthner axon closest to the objective (green), other objects (yellow) are discarded. **(F)** Final segmented Mauthner axon (green) used for measurement. **(G)** Mauthner axon object slices are exported and z-projected to create a 2D planar mask. **(H)** 2D planar mask is polygonised and the polygonal skeleton used to take multiple skeletal width measurements across the length of the detected axon.

Having established imaging parameters that can detect changes to axon diameter at 5 dpf, we next developed a fully automate pipeline to measure of axon diameter. The transgenic line Tg(hspGFF62A:Gal4); Tg(UAS:GFP) that we used to label the Mauthner axon also labels neurons in the posterior lateral line nerve and, on occasion exhibits ectopic fluorescence in other cells (e.g., muscle and skin), as is common for transgenic zebrafish with Gal4:UAS-driven expression (Figs 1A and 2A). Variation caused by ectopic fluorescence expression, as well as autofluorescence, skin pigmentation, and biological batch/clutch variations, lead to an extensive range of inter-image variability and quality. Furthermore, although the VAST system orients the zebrafish in a lateral position with great fidelity, it does not ensure that the midline of the animal runs parallel to the midline of the capillary tubing. Therefore, the trajectory of the Mauthner axon is not always constrained to a predictable number of z-sections in acquired stacks. For these collective reasons, it was not possible to simply convert 3D z-stacks into 2D maximum intensity projections for threshold-segmented analysis. Instead, we employed ML to segment objects of interest in manner that is more agnostic to intensity or position (Fig 2).

Many image analysis platforms, both open-source and commercial, now implement ML or Artificial Intelligence (AI)-based training and segmentation. Here, we employed multiple levels of random forest pixel classification using Arivis Vision4D to generate an automated pipeline for consistent batch measurement of Mauthner axon diameter in live zebrafish.

**Fig 3:**
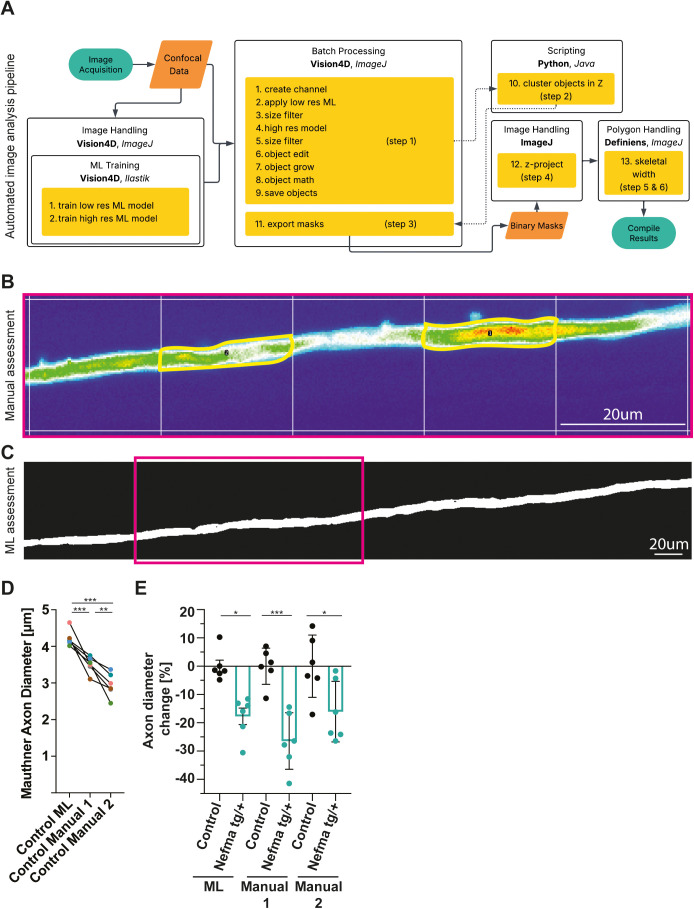
Manual assessment confirms reliability of the machine learning-based quantification of Mauthner axon diameter. **(A)** Workflow diagram illustrates the step-by-step process to create our fully automated image analysis pipeline; the tools used in our study are in bold and alternative suggestions and open-source tools are indicated in italic. All scripts and pipelines can be accessed via Edinburgh Datashare (see Methods). **(B)** Representative thermal look-up table (LUT)-thresholded image of a selected region of a Mauthner axon with an overlaid random measurement grid (yellow, 500 µm^2^ spacing). Manual tracing was performed on a minimum of five grid tiles per sample to calculate mean axon diameter, as described in Methods. **(C)** Representative image of the mask of the complete Mauthner axon in B generated by our machine learning pipeline (ML). Pink box indicates the region shown in B. **(D)** Direct per-sample comparison of Control Mauthner axons, assessed by our automated image analysis pipeline (ML) and manually by two independent assessors (Manual 1 and 2). Each sample is color-matched indicating that Mauthner axon diameter values differ across all 3 measures. (*n* = 5). **(E**) Changes in Mauthner axon diameter for Nefma^tg/+^ were normalized to the respective, assessment-matched controls and calculated in %. Both ML pipeline and manual assessments detect a comparable, statistically significant decrease in Mauthner axon diameter for Nefma^tg/+^ compared to controls. One-way ANOVA with multiple comparison test was used to assess statistical significance. Error bars represent mean with SD. P-values are presented as * *p* < 0.05, ** *p* < 0.01, *** *p* < 0.001. Scale bar = 20 µm. The data underlying this figure can be found in S1 Data.

Single-field imaging of the spinal cord at somite 15–16 was conducted as described above (see also Methods). Selected confocal stacks from multiple runs, encompassing multiple batches, were imported into Vision4D to create a training data set. Each slice of each image was heavily blurred (gaussian, 146 µm radius, alternative software ImageJ) to create an additional low-resolution denoised channel. This channel and the original were used to train a low-resolution random forest pixel classifier model (see Methods) that would detect, very roughly, the Mauthner axons and exclude most artifacts or unwanted signal (highlighted in Fig 2A and 2B, muscles (open arrows), autofluorescence of the skin (solid arrows)). Next, a high-resolution model (see Methods) was trained using only the original channel raw data. Thus, objects outwith the defined size, shape and intensity range were filtered out (Fig 2B–2D, steps 1–9 in workflow diagram Fig 3A, and S1 Video). The imaging, and therefore the segmentation quality, could vary depending on the positioning of the fish and proximity to the objective lens. To account for this, we divided the axons into “top” and “bottom” groups based on their relative z-position, enabling separate analysis of the better-resolved (top) axon. To sort the axon objects into those nearest the objective lens (top) and those furthest away (bottom), relative or relational object filtering was required (e.g., “highest” of two objects with “similar” × centroid values, or group objects with “similar” z centroid). Therefore, a python script was created to cluster objects in the z-dimension and sort the clustered objects into “top” and “bottom” axons (imaged lateral-side-up) (Fig 2E and 2F). This step works partially outside of the image-handling environment and so is represented as an additional process (Fig 3A, dotted arrows).

To obtain an average width measurement for each axon, per-slice binary masks for the top Mauthner axon were exported, projected in Z, and measured in another software package (Definiens DeveloperXD). An example of the process can be seen in Fig 2G and 2H. The workflow diagram (Fig 3A) shows the steps used to create our fully automated image analysis pipeline, states the tools employed in this study (bold text), and suggests alternative or open-source tools that can be used to achieve analogous goals (italic text). A collection of scripts and pipelines for each step are deposited at Edinburgh Datashare (https://doi.org/10.7488/ds/8111).

To test the performance of the automated image analysis pipeline, we used an available transgenic line [52] (here referred to as nefma^tg/+^) that we found to exhibit smaller Mauthner axon diameters and imaged it using our standardized VAST-SDCM settings. We then compared axon diameter measurements obtained from the automated analysis pipeline (ML) with those from fully manual assessments done by two different experimenters (Manual 1 and 2) (see Methods, Fig 3B and 3E). Direct, per-sample comparison of control images showed that Mauthner axon diameter measurements differed between the ML approach and the manual assessors, and between the two manual assessors (Fig 3D). In addition, we observed that manual assessment results in higher variability, together highlighting the importance of an unbiased, automated measurement approach. Notably, independent of ML or manual assessment, we detected a significant ~20% decrease in Mauthner axon diameter in the reporter line compared to controls (Fig 3E). Taken together, we reasoned that our imaging and analysis pipeline is sensitive enough to detect changes in Mauthner axon diameter.

### Chemical screen reveals compounds that change Mauthner axon diameter

After establishing our imaging and analysis pipeline, we next wanted to carry out the screen to identify factors that changed axon diameter. We used the LOPAC^1280^ library because of its well-described compounds, with annotated targets that represent diverse pathways. In addition, it includes a particularly high portion of compounds related to neurotransmission- and ion channel-function. Because it was previously shown that axon diameter can change upon neuronal activity ex vivo [24], we reasoned that this library might enrich for compounds that impact axon diameter growth in vivo.

**Fig 4:**
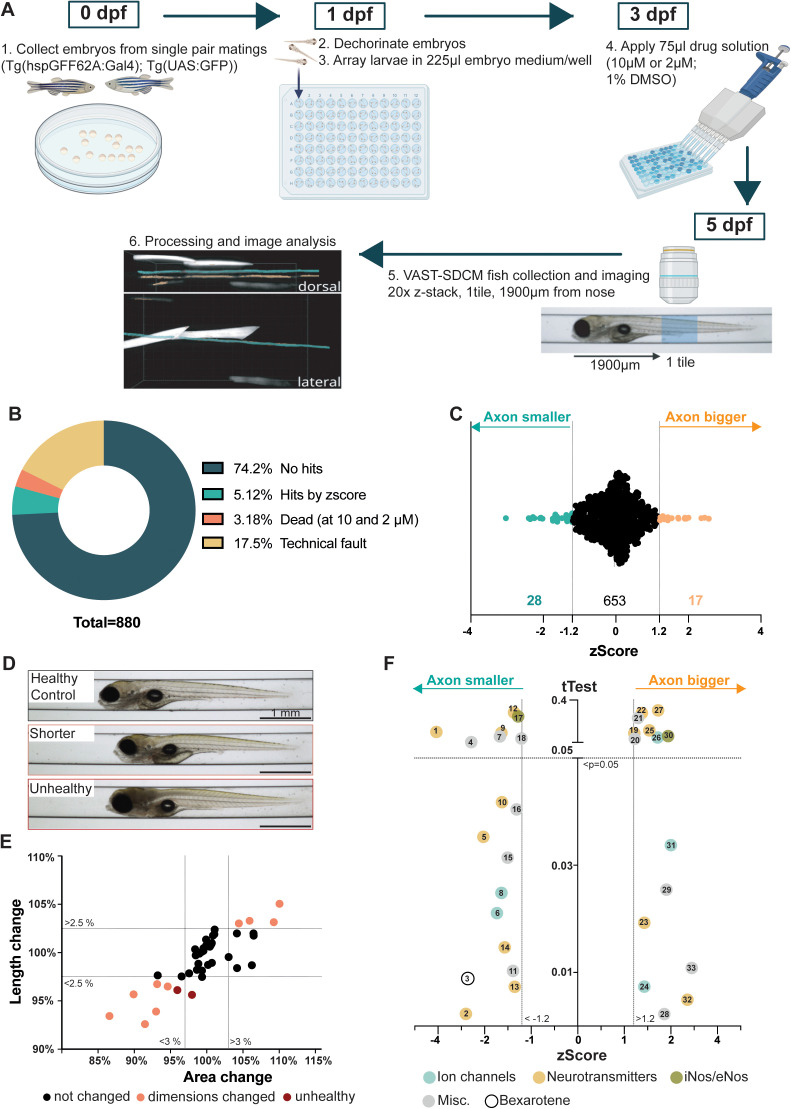
Phenotypic screen identifies compounds that change Mauthner axon diameter. **(A)** Schematic illustrating the workflow of the screening pipeline. **(B)** In the primary screen 880 drug compounds were tested at either 2 or 10 µM for their potential to alter Mauthner axon diameter and approximately 5% were initially identified as hit compounds. A further 3% resulted in death at both dilutions and 17.5% were excluded due to technical issues. **(C)** The z-Score was calculated by comparing each drug with its respective DMSO-treated clutch-specific control. A z-Score of >1.2 or <−1.2 was considered changed in diameter. Twenty-eight compounds decrease and 17 compounds increase Mauthner axon diameter. **(D)** Brightfield images show a healthy appearing control (top), a larva with smaller body dimensions (middle) and one with an unhealthy appearance (bottom). Scale bars = 1 mm. **(E)** Body dimensions (length and area) of each hit compound and their respective DMSO-treated clutch control were subsequently assessed from brightfield images. Changes in over 3% body area and 2.5% length were considered as exclusion criteria (pink). Toxic compounds are indicated in red. **(F)** Results of the primary screen displays hit compounds ranked by z-Score (X-axis) and statistical assessment using Student t test (Y-Axis) comparing each hit compound with its DMSO-treated clutch-specific control. Thirty-three candidate hit compounds are color coded by class of action (turquoise Ion channels, yellow Neurotransmitters; green iNos/eNos species and gray Miscellaneous). In total, 18 drug compounds decrease Mauthner axon diameter beyond z-Score threshold of −1.2 and 15 drug compounds increase Mauthner axon diameter above threshold of 1.2. See also S1 Table for description of compounds. The data underlying this figure can be found in S1 Data. Schematic in A Created in BioRender. Lyons, D. (2026) https://BioRender.com/puyi62e.

For zebrafish screens, compounds in the LOPAC library have commonly been used at around 10 µM to prevent severe impacts on health [53,54]. Since we aimed to treat our larvae at slightly later stages (3–5 dpf), we initially tested 30 randomly chosen drugs at 2, 10, 50, and 100 µM and assessed the survival and health of fish. At 10 µM, we observed little impact on health and survival of larvae (~1% mortality) and thus selected 10 µM as the primary concentration for each compound. Compounds that did cause larval death at 10 µM were rescreened at 2 µM in the primary screen. To maximize our throughput and test more compounds per 96-well plate, we placed 3 fish/well which allowed testing of 30 drugs (3 wells per drug for total of 9 animals per compound plus 6 wells of controls: each 96-well plate of 3 animals per well was imaged in ~5 hours. To accommodate for clutch-specific differences in axon diameter and for any changes that may occur to diameter over the imaging period, we included 3 wells with 3 fish/well treated with 0.5% DMSO as clutch-specific controls, spread out across the 96-well plate. In the screen, animals were treated with compounds from 3 to 5 dpf, imaged at 5 dpf and assessed using our automated analysis pipeline (Fig 4A). To assess how test compounds influence axon diameter, we normalized compound effects on axon diameter relative to controls and calculated a z-score to rank how compounds differed from controls. We did so by comparing axon diameter measurements for each drug-treated sample to those of the corresponding DMSO-treated clutch and plate-specific control (see Methods for calculations). This standardization is common practice in high-throughput drug screening approaches because it accounts for variability across wells, plates and batches and allows ranking of the impact the identified hits have [55]. Given that we image at subcellular resolution and aim to detect changes as small as 10% in an axon as big as the Mauthner axon, we set a threshold z-score >+1.2 or <−1.2 to define hit compounds in the primary screen. To minimize technical, imaging, or analysis-related errors we manually inspected the z-stacks and masks for each candidate hit as well as their respective controls. In some cases, imaging errors (e.g., drift of fish in the z-axis) led to incorrect detection of the Mauthner axon. Therefore, we excluded any data points without accurate axon identification and mask-based measurement from our analysis (For a detailed description of each step, see Methods).

In total, we tested 880 drugs for their ability to affect axon diameter. We found that 28/880 drugs (3.18%) were lethal at both 10 and 2 µM dilution and 154/880 treatments (17.5%) had to be excluded due to technical difficulties (low image quality, false detection of the Mauthner, *n* number <4). Of the remaining compounds, 653 (74.2%) did not meet the z-score threshold, while 45/880 compounds (approximately 5%) were classified as hit compounds (Fig 4B and 4C).

**Fig 5:**
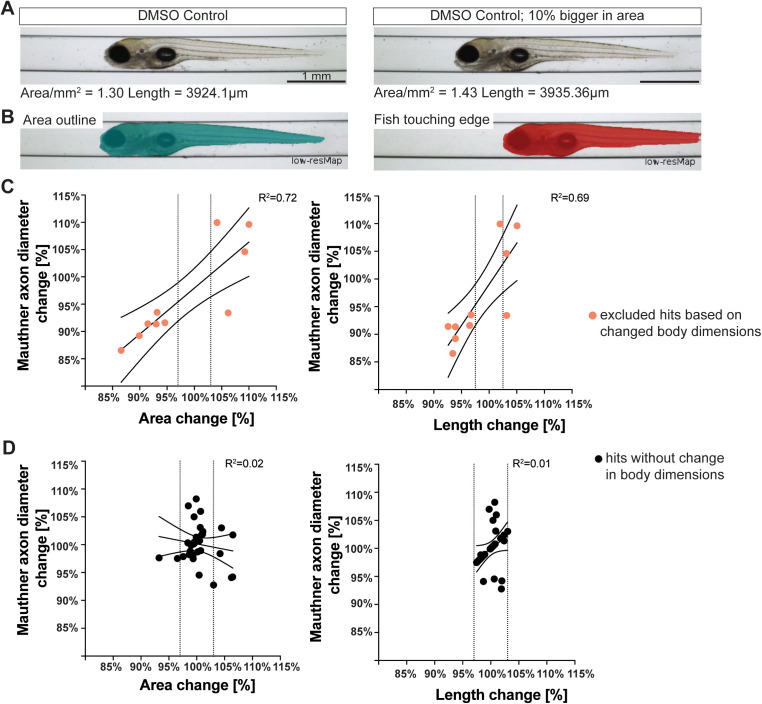
Assessment of exclusion criteria for body dimensions. **(A)** The two depicted brightfield images acquired on VAST-SDCM of DMSO-treated controls vary by 10% in area which we cannot manually assess by eye. Scale bars = 1 mm. **(B)** Body dimension (length and area) was calculated on brightfield images from VAST-SDCM by training a machine learning classifier in DeveloperXD. Exclusion criteria (3% area change and 2.5% length change) were estimated based on averaged variations across different DMSO-treated controls (See Methods for details). **(C)** Hit compounds that were excluded based on body size are shown in pink. For those, both area and length correlate with axon diameter (*R*^2^ = 0.72 area and *R*^2^ = 0.69 length). **(D)** Hit compounds without change in body dimensions are shown in black (right) and have no correlation between area or length and axon diameter (*R*^2^ = 0.02 area and *R*^2^ = 0.01 length). The data underlying this figure can be found in S1 Data.

To check for the impact each candidate hit might have on the health of the fish, we manually assessed all accompanying brightfield RGB images acquired by VAST, for any discoloration (sign of ill health) or deformations. We excluded 2 compounds because fish were discolored or their spine slightly deformed (Fig 4D). Next, we wanted to make sure that the remaining 43 hit compounds did not influence axon diameter secondary to impacting the overall size of the fish. To exclude candidate hits that primarily impact body dimensions, we measured body area and length of all animals treated with candidate hits and their respective controls. Determining relatively subtle changes to animal size (e.g., on the order of 10%, Fig 5A) by eye is very challenging so we applied further automated methods. This time, we trained a random forest pixel classifier to recognize whole fish larvae (Fig 5B). We measured body area and length using measurements built into DeveloperXD as described in Methods (Object Area, Length (bounding box)). To define exclusion criteria, we first assessed variation in whole-body area and fish length. We did so by pooling the values for area and length of DMSO-treated controls across various clutches and imaging rounds and calculating their mean values, and standard deviation. A single standard deviation from the mean length of all controls (3.93 mm) was 2.25% and of area (1.41 mm^2^) was 2.92%. Therefore, we considered changes exhibited by hits that were greater than a single standard deviation as likely affecting animal size and thus set a change of both 2.5% in length and 3% area as an exclusion threshold for altered body dimensions (see Methods; stippled lines Fig 4E). This resulted in the exclusion of 10 hit compounds (Fig 4E). Validating these exclusion criteria, we found that changes in body area and length were significantly correlated with axon diameter for the 10 excluded hits (*R*^2^ = 0.72 for area and *R*^2^ = 0.69 for length) (Fig 5C). In contrast, there was no correlation between axon diameter and body area or length for the remaining 33 candidate hits (*R*^2^ = 0.02 for area and *R*^2^ = 0.01 for length) (Fig 5D), suggesting alteration to axon diameter independent of body size.

Among the 33 remaining hit compounds, 18 decreased Mauthner axon diameter, while 15 increased it (Fig 4F and S1 Table). Notably, 19 of the overall 33 hit compounds are linked to neurotransmission and ion channel function. This enrichment is consistent with the LOPAC library composition, in which ~60% are neurotransmission and ion channel-related compounds. In contrast, other compounds target nitric oxide species, lipid signaling, phosphorylation, cytoskeleton, and extracellular matrix. To complement the z-score ranking, we performed a Student *t* test comparing each hit compound exclusively to its respective DMSO-treated control, enabling standardized effect size assessment and to serve as guidance which compounds to prioritize for validation. This analysis identified 18 out of 33 compounds that statistically significantly alter axon diameter. S1 Table highlights all hit compounds, including their ranking metrics and action class.

In summary, we performed the first in vivo discovery screen designed to identify compounds that modulate axon diameter. Out of 880 compounds tested, following rigorous data curation, we identified 33 compounds that influence Mauthner axon diameter growth. We provide our complete list of hit compounds to the neuroscience community as a resource to facilitate further investigations.

### Validation of hit compounds reveals that beta-2-adrenoceptor agonists and dopamine antagonists increase Mauthner axon diameter growth

Next, we wanted to validate hit compounds from our primary screen. We first assessed the action class of compounds and prioritized hit compounds that might influence axon diameter growth by modulating neuronal activity. In addition, we prioritized compounds according to (1) their z-score and effect size rank; (2) repeated appearance of their class of action amongst hits; (3) presence of both antagonist and agonist of the same class that introduce opposing effects on diameter; and (4) their ability to increase the diameter of the already large Mauthner axon. Based on these triage steps, we first focused on compounds linked to adrenoceptors (2 antagonists decreasing and 1 agonist increasing diameter) and dopamine signaling (2 antagonists increasing axon diameter).

**Fig 6:**
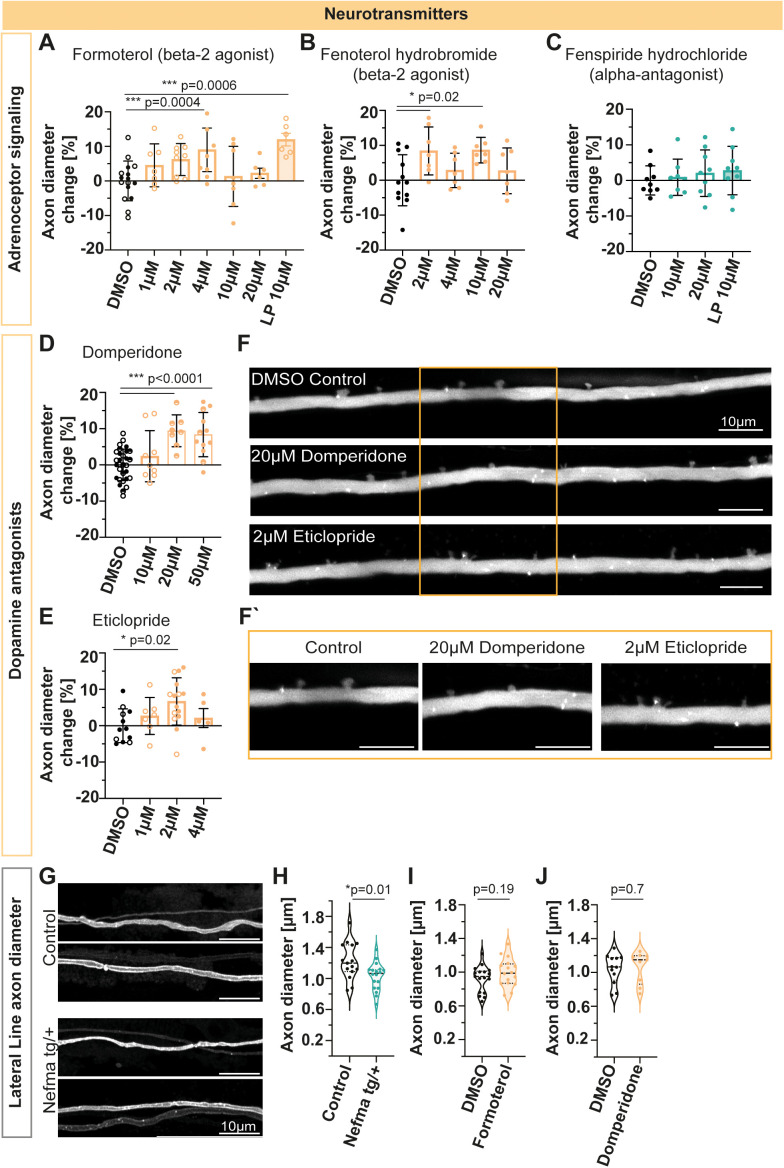
Beta-2-adrenoceptor agonists and dopamine antagonists increase Mauthner axon diameter growth. Validation of selected hit compounds different vendors was performed at different concentrations (1–50 µM) and compared to control fish treated with 0.5%–1% DMSO. Fatal or health-impacting dilutions are excluded. The change of Mauthner axon diameter for each drug was normalized to the clutch-specific DMSO-treated control and calculated in %. If available, the original LOPAC^1280^ drug was tested at its original dilution (LP). **(A)** Beta-2 Adrenoceptor agonist Formoterol resulted in a significant increase in Mauthner axon diameter at 4 µM and when testing the original LP dilution at 10 µM. **(B)** A second beta-2 adrenoceptor agonist, Fenoterol hydrobromide shows an increase in Mauthner diameter at 1 and 4 µM. **(C)** Alpha Adrenoceptor antagonist Fenspiride hydrochloride did not show the initially detected decrease in Mauthner axon diameter, no significant change was detected. **(D, E)** Both Dopamine antagonist Domperidone and Eticlopride resulted in a significant increase in Mauthner axon diameter. **(F–F′)** Fish were imaged using 40 × objective at a Zeiss LSM880 with AiryScan FAST mode with 1.8 zoom for validation studies. Exemplified confocal images and zoom in (F′) visualize increase in Mauthner axon diameter when fish were treated with Dopamine antagonists Domperidone and Eticlopride. **(G)** Exemplary images for PLLn axons comparing control and Nefma^tg/+^ larvae. **(H)** PLLn axon diameter is significantly decreased in Nefma^tg/+^ transgenic line compared to control. **(I, J)** Both, Formoterol (I) and Domperidone (J) did not show a statistically significant change in PLL axon diameter but indicate a trend towards increased diameters for Formoterol. If applicable, individual clutches are shown in open vs. closed circles. N number indicated by individual data points in all graphs. One-way ANOVA followed by Dunnett`s multiple comparison test (A–E) or Student *t* test (H–J) was used to assess statistical significance. Multiple adjusted p-values are shown in each figure panel and are presented as * *p* < 0.05, ** *p* < 0.01, *** *p* < 0.001. Error bars represent mean with SD. Scale bars = 10 µm. The data underlying this figure can be found in S1 Data.

We ordered the same compounds or those that target the same pathway from different suppliers. To assess dose-dependent effects of the new compounds on Mauthner axon diameter, we performed serial dilutions ranging from 1 to 100 µM and manually assessed compound toxicity by eye to determine which concentrations induced developmental deformities or lethality of larvae. We excluded these concentrations from further analysis. When available, we re-tested the original LOPAC library compound at the concentration we used in our initial screen (10 or 2 µM). While the VAST-SDCM system and our imaging pipeline enabled us to perform the large-scale primary screen, our validation experiments did not require high-throughput imaging. To achieve higher resolution imaging, we used our previously established method for imaging axon diameter by confocal super-resolution imaging (Figs 1D, 6F, and 6F′). We imaged Mauthner axons of drug-treated and DMSO-treated control larvae and used a semi-automated script to measure axon diameter, given that our ML-driven image analysis was bespoke to images acquired on our VAST-SDCM platform. To account for clutch-specific variability we normalized axon diameter by calculating the change relative to DMSO-treated controls.

Our validation tests revealed that the beta2-adrenoceptor agonist Formoterol increases the axon diameter of the Mauthner at 4 µM (~8%), with the original LOPAC^1280^ compound also showing a significant increase (~11%) in diameter at 10 µM (Fig 6A). To assess another beta-2 adrenoceptor agonist, we applied Fenoterol hydrobromide and detected a significant increase in axon diameter at 2 and 10 µM (~8% to 9%, Fig 6B). We did not detect changes in Mauthner axon diameter when applying the alpha-adrenoceptor antagonist Fenspiride hydrochloride, which decreased axon diameter in the initial screen (Fig 6C). We were not able to acquire the third adrenoceptor-related compound A315456 on the market. Next, we assessed the two hit compounds targeting Dopamine signaling: the dopamine antagonists Domperidone and Eticlopride. Both compounds significantly increased diameter between 6% and 10% in our validation studies (Fig 6D–6FFigure 6EFigure 6F′).

Next, we wanted to know if these hit compounds influenced the diameter of other axons, particularly those of smaller diameter than that of the Mauthner neuron. The transgenic line Tg(hspGFF62A:Gal4) used throughout this study also labels neurons of the posterior lateral line (PLLn) nerve of the peripheral nervous system. To test whether we could detect changes in the diameter of PLLn axons, we used the Nefma^tg/+^ line and observed a significant ~22% decrease compared to controls (1.226 µm control and 1.027 µm Nefma^tg/+^, *n* > 15). When applying Formoterol or Domperidone, we did not detect a statistically significant difference in PLLn axon diameter (Fig 6I and 6J). Nonetheless, mean values for the Formoterol dataset were 0.921 µm for DMSO-treated controls and 0.997 µm for Formoterol-treated larvae. This mean difference of approx. 70 nm reflects an 8% percent increase, very close to the one observed for the Mauthner axon (8.9%; Fig 6A) suggesting a trend towards increased axon diameter with beta-2 adrenoceptor agonist Formoterol. See data source file for considerations for power calculations for the detection of subtle changes to axon diameter.

### Hit compounds exhibit complex influences on different neuronal compartments

**Fig 7:**
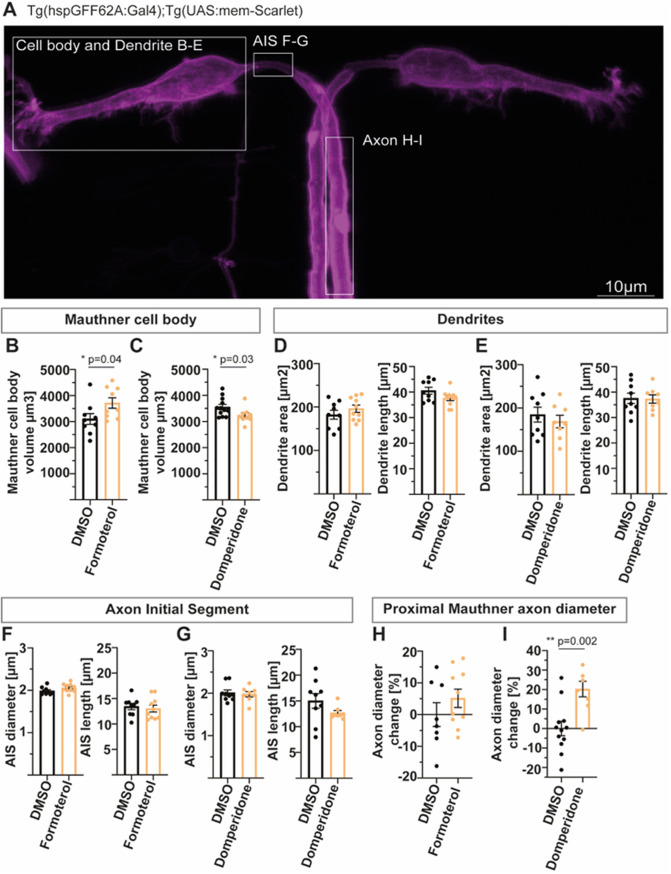
Hit compounds exhibit complex influences on different neuronal compartments. Morphological analyses of multiple compartments of the Mauthner neuron (cell body, dendrite, AIS and proximal axon) were performed in larvae treated with beta-2-adrenoceptor agonist Formoterol (4 µM) or Dopamine antagonist Domperidone (20 µM) and compared to clutch-specific, DMSO-treated controls. **(A)** Mauthner neurons and respective domains were imaged in the hindbrain from a dorsal view using the membrane-labeling transgenic line Tg(hspGFF62A:Gal4);Tg(UAS:mem-Scarlet). **(B)** Beta-2 Adrenoceptor agonist Formoterol resulted in a significant increase in Mauthner cell body volume. **(C)** Dopamine antagonist Domperidone resulted in a minor but significant decrease in Mauthner cell body volume. **(D, E)** Both, beta-2 Adrenoceptor agonist Formoterol (D) and Dopamine antagonist Domperidone (E) did not change dendrite area or length. **(F, G)** No significant changes were detected for AIS diameter and length for both, beta-2 Adrenoceptor agonist Formoterol (F) and Dopamine antagonist Domperidone (G). **(H)** Formoterol resulted in a non-significant trend towards increased proximal Mauthner axon diameter, whereas **(I)** Domperidone resulted in a significant increase in proximal Mauthner axon diameter. *N* number is indicated by individual data points in all graphs. Student *t* test was used to assess statistical significance. Multiple adjusted *p*-values are shown in each figure panel and are presented as * *p* < 0.05, ** *p* < 0.01. Error bars represent mean with SD. Scale bars = 10 µm. The data underlying this figure can be found in S1 Data.

Live imaging in zebrafish enables assessment of the morphology of entire neurons in vivo. Thus, we tested how our primary hit compounds affected Mauthner cell body size and dendrite morphology, axon initial segment (AIS) length and diameter, as well as the diameter of the most proximal domain of the axon after the AIS. To do so, we used a transgenic line that labels neuronal and axonal membranes (Tg(hspGFF62A:Gal4); Tg(UAS:mem-Scarlet), Fig 7A), applying one drug per compound class at the concentration where we observed the strongest influence on Mauthner axon diameter at somite 16.

For Formoterol (4 µM) we found a significant increase in Mauthner cell body volume (Fig 7B), but no change in dendrite area or length (Fig 7D), no change in AIS diameter or length (Fig 7F) and a trend increase in the diameter of the most proximal area of the axon after the AIS (Fig 7H), in line with the significant increase in axon diameter in the more distal portions of the axon examined in our screen and initial validation. In contrast, treatment with Domperidone (20 µM) revealed more complex alterations to neuronal and axonal morphology, with a mild but significant decrease in Mauthner cell body size (Fig 7C), no changes in dendrite area and length (Fig 7E), or AIS diameter and length (Fig 7G), but a 22% increase in the diameter of the proximal axon domain after the AIS (Fig 7I).

Taken together, we have validated two hit classes, beta-2 adrenoceptor agonists and dopamine antagonists, as affecting Mauthner axon diameter growth. The fact that our screen was successful in identifying relatively subtle changes to axon diameter exemplifies the possibility of using zebrafish for in vivo screens to identify regulators of subcellular features of cell morphology.

## Discussion

Zebrafish are a powerful model for in vivo discovery screens, with ongoing innovation in biosensors, reporters, imaging and image analysis, now making it possible to investigate previously intractable processes [48,53,54,56–58]. Here, we have brought the power of scalable high-resolution high-content imaging of zebrafish to bear on the biology of axon diameter, a largely overlooked, but essential feature of neuronal morphology and neural circuit structure and function.

### Technical considerations

A major constraint on screening for changes to sub-cellular morphological parameters, such as axon diameter, is balancing the resolution required to detect meaningful phenotypes and the time required to capture the images to do so. To screen for potentially sub-micrometer scale changes in axon diameter, we used the Mauthner neuron [45–47] as a model, because its axon grows to ~5 µm in diameter by 5dpf [20,44] meaning that changes could be resolved using our automated confocal screening system (VAST-SDCM). We tested 880 compounds for their potential to alter axon diameter using a robust automated analysis pipeline.

In the future, it will be important to extend discovery screening to better understand how alterations to axon diameter relate to other features of neuronal morphology. For example, as part of our validation studies, we assessed how hit compounds affected a variety of additional factors such as cell body size and dendrite morphology relative to axon diameter, highlighting also the potential to screen for many features of neuronal morphology at once. Indeed, the assessment of neuronal morphology could be complemented by parallel analyses of molecular components of neurons and axons, e.g., through the availability of ever-increasing suites of transgenic reporters. In addition, and as we have previously noted, zebrafish allow assessment of changes to the diameter of significantly smaller axons, which will be important to interrogate in future discovery studies. Our reported implementation of our VAST-SDCM system had a relatively limited resolution of 0.286 µm/X–Y pixel, best suited to detecting changes to the large Mauthner axon. To be able to perform robust scalable analyses of additional fine features of neuronal morphology or assess different neurons with smaller-diameter axons in vivo will require further innovation in microscopy focused on improving the sensitivity and resolution of image acquisition. For example, our imaging to validate hit compounds used super-resolution confocal live imaging with 0.054 µm X–Y pixel dimensions, which if possible to achieve at higher speeds, could facilitate higher content analyses.

In addition to requiring high-resolution fast imaging scalable discovery screens necessitate efficient data acquisition and analysis pipelines. As noted, in our reported screen we acquired single 3D z-stacks per animal from VAST-SDCM, which took ~5 hours per plate to screen 30 compounds per plate (*n* up to 9 per compound plus controls), meaning that screening ~1,000 compounds requires ~33 such imaging sessions. In principle, alternative high-speed image acquisition solutions could be applied to discovery screens, such as light sheet microscopy. However, it is important to note that one requires image handing and analysis pipelines capable of efficiently dealing with high volumes of complex data. For example, running the automated image analysis pipelines associated with our screen was very time-consuming. We carried out our initial analyses on a standalone PC workstation, which required ~15 min per image stack and so ~2,500 hours to process and analyze the ~10,000 confocal z-stack images acquired through our screen. We have recently tested our image analysis pipeline using a newly acquired dedicated hub running the Arivis software, which reduced processing time to ~5 min per imaging stack. Therefore, going forward screening ~1,000 compounds would take ~800 hours of image processing. Although such automated image analyses remain time-consuming, as machine learning-based processes advance and become more accessible, image analysis pipelines are likely to become more efficient.

A key consideration in the execution of screens is false-positive and false-negative hit discovery. Our screen identified 33 hit compounds that changed Mauthner axon diameter. Our validation studies confirmed the effects of some hit compounds (discussed below), but also revealed false positive hits, a common occurrence in discovery screens. In the context of our screen, additional points are noteworthy:

1. Identifying changes to axon diameter placed constraints on the effect sizes that we were likely to observe through our screen. Unlike screens that aim to identify the presence or absence of a cell type or structure of interest, our screen aimed to identify relatively small changes to a one-dimensional parameter. Here, we aimed to enrich for discovery of factors with specific effects on axon diameter growth and thus only treated animals with compounds between 3 and 5 dpf to assess their effects at a period after axons had fully grown in length. Although the Mauthner axon grows over this 2-day period, it does so by ~1.5 µm in diameter in the region that we examined in our screen [20]. This limits the absolute effects that compound treatments could exert over this time. Furthermore, we excluded drugs that affected general animal growth/size in our screen, which might have affected axon diameter more severely, e.g., by influencing overall growth or animal health. Furthermore, it is not clear to what extent the diameter of the Mauthner axon can increase its rate of growth over these stages, meaning that the relatively small effect sizes that we documented may reflect a ceiling to how quickly the axon can grow in diameter over this time.
2. Although the morphology of the Mauthner axon is highly stereotyped, its diameter does exhibit variability on the order of ± 10% between animals, even within the same clutch. Given the relatively small effect sizes detectable through our screen, this variability increased the n number required to detect phenotypes with a high degree of confidence. We had calculated an “*n*” of 9 animals per condition as providing sufficient power to detect subtle changes in our screen. Although the VAST-SDCM is generally efficient in delivering and imaging animals, many samples processed during our screen were discarded such that the n number for certain compounds was lower than hoped for, likely contributing to both false positive and negative outcomes. For example, we experienced 17.5% technical exclusions. These resulted from drift of samples in the imaging capillary on the VAST-SDCM system during acquisition, decreasing image quality, which cannot be tolerated when screening for changes to fine morphological features. In addition, our image analysis pipeline was established to be sensitive to blurred features of the Mauthner axon and would exclude these from consideration. Also, due to the nature of the transgenic reporter, certain clutches of fish exhibited higher ectopic fluorescence from other cells, which, if influencing Mauthner detection or mask generation, led to sample exclusion. Future screens focused on detecting changes to sub-cellular morphology will benefit from increasing the number of animals to ensure that the required “*n*” passes through the entire analytical pipeline. Solutions such as the generation of targeted knock-ins of reporters to endogenous loci will minimize variability that can be associated with transgenesis-based reporters [59].
3. Chemical screens can be subject to variability due to deviation from expected compound concentrations, plate effects such as evaporation and edge effects. It is particularly challenging to determine chemical compound concentrations within the tissues of larval zebrafish in a screen context, and it is important to note that dose–response activities of compounds in vivo can exhibit complex non-linear effects.

These general caveats of primary screens carried out in vivo mean that rigorous validation steps, as we have outlined are required. Nonetheless, our screen and validation pipeline has already confirmed hit compounds that do affect axon diameter, namely those affecting dopamine signaling and adrenoreceptors.

### Biological considerations

Domperidone and Eticlopride are both D2-dopamine receptor antagonists and were found to increase Mauthner axon diameter. Domperidone has been shown to affect locomotor behavior in zebrafish with D2-receptor antagonism associated with increased neuronal excitability across vertebrates [60–62]. Interestingly, dopaminergic neurons have been shown to modulate the sound-evoked Mauthner cell-mediated C-startle response [63]. This suggests the possibility that systemic dopaminergic manipulation may influence the activity of the escape circuit, including Mauthner neuron activity, which may in turn influence axon diameter. In support of the premise that neuronal activity might influence axon diameter is evidence from ex vivo preparations of hippocampal neurons which showed that axons can locally widen upon high-frequency stimulation [24]. However, our extended analyses of the effects of Domperidone pointed to complex effects of the compound on neuronal morphology, whereby while axon diameter was enlarged, the size of the Mauthner cell body was reduced. Therefore, disentangling the mechanisms by which axon diameter and cell growth are regulated with respect to one another in general and in the context of regulation of dopamine signaling will require further in-depth interrogation. Our studies highlight the importance of taking a holistic approach to interrogating the effects of factors on neuronal morphology as a whole.

Formoterol and Fenoterol hydrobromide are both beta2 adrenoceptor agonists that increased Mauthner axon diameter. Beta-2 adrenoceptors are G protein-coupled receptors that mediate various physiological processes that can be activated by endogenous catecholamines like noradrenaline and adrenaline in the nervous system and influence behavior [64–66]. However, they also have effects within and outwith the nervous system playing prominent roles in smooth muscle relaxation and subsequent vasodilation [67,68]. In contrast to the differential effects of D2 acting compounds on Mauthner cell body and axon size, the beta2 adrenoceptors exhibited general increases in neuronal size, both of the Mauthner cell body and its axon, and trending to do so also in the neurons of the lateral line nerve in the PNS, suggesting they may influence general neuronal growth pathways. In addition to deconstructing how our currently validated hits affect axon diameter, future studies will be required to test and confirm the activity of the other hits from our screen. This will expand our understanding of the many mechanisms likely to influence axon diameter, including those suggested by our screen, such as lipid signaling, pathway-specific phosphorylation, and ECM/cytoskeletal regulation.

Our study represents the first dedicated effort to discover the molecular basis of axon diameter growth. Follow-up studies will need to characterize the effect of hit compounds on different specific neurons of the CNS and PNS that have axons of distinct sizes, and at distinct stages of system and circuit maturation. In addition, interrogation of the molecular mechanisms underlying compound-induced effects will require complementary genetic perturbations of candidate pathways. Here, one important consideration will be the choice of genetic manipulation used to assess the roles of candidate targets of hit compounds. This may require conditional (spatial and temporal) activation or inhibition of candidate targets of hit compounds in vivo, which is increasingly tractable using zebrafish as a model system [69,70]. In addition, it will be important to extend discoveries made in zebrafish to mammals, including human models, both in vitro and in vivo. In summary, our screen provides a foundation and resource for the community to help further understanding of the biology of axon diameter.

## Methods

### Ethics statement

Adult zebrafish were housed and maintained in accordance with standard procedures in the Queen’s Medical Research Institute zebrafish facility at the University of Edinburgh. All experiments were performed in compliance with the UK Home Office, according to its regulations under project licenses PP5258250 and PP3290955.

### Zebrafish husbandry and transgenic lines

Adult zebrafish were subject to a 14/10 hours, light/dark cycle. Embryos were produced by pairwise matings, collected within 2 hours post-fertilization (hpf) and raised with 50 embryos per dish at 28.5 °C in 10 mM HEPES-buffered E3 Embryo medium or conditioned aquarium water with methylene blue. All experiments used zebrafish larvae up 5 dpf on a wild type (AB/WIK/TL) or nacre^−/−^ [71] background. At these ages, sexual differentiation of zebrafish has not yet occurred. The following transgenic lines were used for this study: Tg(hspGFF62A:Gal4) [49,72], Tg(UAS:GFP) [49], Tg(UAS:mem-Scarlet) [20], Tg(UAS:memGFP) [49], Tg(nefma-mcherry) [52]. When characterizing the Tg(nefma-mcherry) transgenic reporter (here referred to as Nefma^tg/+^), we noted a reduction in Mauthner axon diameter in the heterozygous form and used it as a model for smaller axon diameters. Controls refer to clutch-specific siblings.

### Hardware setup (VAST-SDCM platform)

All animals in the primary LOPAC screen were imaged using the VAST-SDCM platform described in Early and colleagues [48]. Briefly, three fish were loaded into each well of a 96-well plate and brought to a CSU-X1 spinning disk (Yokogawa) unit with dual AxioCam (Zeiss) 506 mounted on an Examiner frame (Zeiss) using a combination of LP Sampler and VAST robotic fluidics (Union Biometrica). We used a W Plan-Apochromat 10×/0.5NA M27 75 mm dipping lens (Zeiss) and generated 6 stitched z-stacks for whole fish overview images (Fig 1A). Otherwise, for axon diameter measurements, the system was configured to acquire a single z-stack 1,900 µm distal from the tip of the larvae head using a W Plan-Apochromat 20×/1.0NA Corr DIC M27 75 mm objective lens (Zeiss). The single 333 × 249 × 300 µm (XYZ) stack with 2 × 2 binning (286 nm pixels) and 300 ms exposure was used for optimal speed while maintaining signal:noise ratio for image processing.

### Chemical screen: Larvae preparation and compound treatments

Out-crosses of the Tg(hspGFF62A:Gal4);Tg(UAS:GFP) transgenic line to nacre fish were used for the primary screen. Embryos were collected within 2 hpf and raised with 50 embryos per dish. Individual clutches were separately analyzed to reduce variability throughout. Embryos were enzymatically dechorionated at 24–30 hpf using protease from *Streptomyces griseus* (0.5 mg/mL for 6 min) (Sigma-Aldrich, St. Louis, MO) and washed with E3 media. Afterwards, three embryos per well were manually arrayed into 96-well plates in 225 μL E3 media. At 2 and 3 dpf, larvae were checked for viability; unhealthy animals were manually removed and replaced in equal volumes with clutch-matched larvae. First, 10 mM compound stocks in DMSO (LOPAC^1280^, Plate 1–11; Sigma-Aldrich, St. Louis, MO) were serially diluted using a multi-channel pipette to 2 mM, 100% DMSO and frozen at −80 °C. On the day of drug treatments, a 4× concentrated treatment solution was generated by diluting 5 or 1 µl in 245 µl E3 media (40 and 8 µM, respectively, 2%–0.4% DMSO). Between 70 and 75 hpf, 75 µL of this 4× stock was added directly into the larval wells for a final concentration of 10 or 2 μM in 0.5%–0.1% DMSO). Each compound was tested on 3 wells of 3 fish per well (*n* = 9) and compared against 0.5% DMSO-treated negative controls of the same clutch (*n* = 9–12) within the same plate. Treatment plates were incubated under standard temperature conditions for 2 days without compound refreshment. At 5 dpf, larvae were anaesthetized with 600 µM tricaine before imaging. One 96-well plate with 30 compounds and respective controls was imaged within 4.5–5.5 h. The control wells were spread out over the plate and/or imaged throughout the day to spread control variability due to development while imaging live animals over time.

### Automated image analysis pipeline

A machine learning training set that covered the range of sample and image variation encountered in the study was created in Arivis Vision4D v3 from full XYZ stacks in CZI format acquired from 9 independent imaging runs. Two random forest pixel classifiers were created using the “Machine Learning Trainer” feature: LowResML and HighResML. Both were set to use 100% scaling as well as all features at all pixel sizes. LowResML was trained using both gaussian-blurred and original raw data channel and with classes axon, fuzzy, and Background. HighResML was trained with only the original unprocessed data channel and with classes axonHighRes, fuzzyHighRes, muscleHighRes, and background. Training samples were annotated on 37 images in this data set as follows: LowResML - Axon class (71 samples, positive class), Fuzzy class (271 samples, negative class), Background class (139 samples, negative class); HighResML—AxonHighRes class (88 samples, positive class), fuzzyHghRes class (461 samples, negative class), muscleHighRes class (72 samples, negative class), background class (166 samples, negative class). Both models were required sequentially to successfully detect target Mauthner axons at full resolution. LowResML was used to detect target Mauthner regions and exclude most of the skin autofluorescence and ectopic muscle GFP expression (Fig 2A and 2B). Following size and shape filtering of low-resolution segmented objects (Fig 2C), highResML was applied (Fig 2D). High-resolution segmented objects were only kept if they were of the correct AxonHighRes class and were contained within the low-resolution Axon class (Fig 2E). High-resolution axon segments were then sorted, using a Python script linked to Vision4D, into upper, lower, and solo Mauthner axons (Fig 2F). Single-plane binary masks were exported and z-projected in FIJI before being imported into Definiens DeveloperXD for measurement of polygonal skeletal width.

The detailed workflow is comprised of the following steps and depicted in Fig 3A:

1. Create additional gaussian channel
2. Apply low-resolution ML model
3. Filter objects for size (>16,000 µm^2^)
4. Apply high-resolution ML model
5. Filter objects for size (>13,000 µm^2^)
6. Fill inclusions (fill holes) in low-res objects
7. Dilate low-resolution objects (5 pixels)
8. Object math: intersect low-resolution objects with high-resolution objects
9. Keep only intersected objects
10. Sort objects into Z-dimension clusters and keep top-Z clusters (integrated python script)
11. Export masks of remaining Mauthner objects slice-wise
12. Z-project binary masks to 2D image
13. Import, segment, and measure binary masks

All scripts and pipelines for each step are deposited at Edinburgh Datashare (https://doi.org/10.7488/ds/8111).

After individual values for Mauthner axon diameters were measured a python script was used to filter and sort axon diameter values according to image day, drug treatment and clutch. Afterwards, the Z-Score was calculated to rank drugs and detect each drug’s effect on Mauthner diameters of drug-treated fish. The following equation was used


$$\begin{array}{c} Z-Score=\;(x-\mu )/\sigma . \end{array}$$

Here, *x* is the mean of the measured axon diameters from each drug; *µ* is the mean of the axon diameter from the clutch and plate-specific, DMSO-treated control and *σ* is the standard deviation of the respective control group. A z-score >+1.2 or <–1.2 was defined as hit compound in the primary screen. Afterwards, Mauthner axons detected in Vision 4D and axonal mask from Definiens for each hit compounds and respective control were manually assessed for (1) optimal imaging (for example, no drift in z), (2) correct detection of the Top Mauthner and (3) correct mask generation. If any of them did not guarantee optimal detection and tracing of the Top Mauthner axon the values were excluded. If for a given compound the *n*-number is lower than 4, we excluded that compound as well. All excluded compounds fall under the category technical difficulties (Fig 4B).

### Whole zebrafish size dimensional analyses

Color photographs from VAST-SDCM were used to assess the health and overall dimensions of fish treated with hit compounds according to z-Score. Images of each fish were imported into Definiens DeveloperXD image analysis software (Definiens AG). A montage was created from 20 images and used to train a random forest pixel classifier to detect fish photographed inside the capillary tube. The trained machine learning classifier was used in an automated DeveloperXD pipeline to detect all fish, exclude those touching the edges of the photograph, and then measure the Area and Length (bounding box) of each fish using built-in functions with those names. The overall appearance of the fish was assessed manually.

To estimate variations in length and area, both values of DMSO-treated control fish across 14 different clutches and 10 individual VAST-SDCM runs were averaged. We considered this spread in controls as the normal change in dimensions. For exclusion criteria, the single standard deviation from the mean area change (2.92) and length change (2.25) were calculated. Changes greater than the single standard deviation from the control values for both area and length were considered to change animal size and thus exclusion criteria were set to >3% change in area and >2.5% change in length. If a given hit compound fell beyond both criteria, it was considered as changed in body dimensions and excluded (10 hits in total). We performed a Pearson correlation to check for the validity of our exclusion criteria.

### Validation of candidates

To validate hit compounds from the primary screen, the same compounds were ordered from different companies (Tocris, Cambridge Bioscience, Sigma, Santa Cruz, Cayman Chemicals). Embryos were again plated with 50 embryos per dish and were dechorinated. Depending on the price and amount of drug available, fish were treated in either 50 per dish or 96 wells with three fish per well. Clutch-specific DMSO-treated controls were included in every experiment and handled the same way as drug-treated fish. If the original LOPAC^1280^ compound was still available, it was included and used as a positive control at its original dilution. Drugs were serially diluted according to the dilution used in the primary screen, ranging from 1, 2, 4, 10, 20, 50, to 100 µM. Treatment started either at 1 or 3 dpf with daily compound exchanges. Dilutions that resulted in unhealthy fish are excluded. Only healthy-appearing fish with inflated swim bladders were subsequently imaged using high-resolution FAST imaging on a Zeiss LSM880 Airyscan confocal microscope.

### Live in vivo imaging

For all our validation experiments, we used Tg(hspGFF62A:Gal4);Tg(UAS:GFP), except in Fig 6G and 6H when we used Tg(nefma-mcherry); Tg(hspGFF62A:Gal4);Tg(UAS:mem-GFP). For assessments in Fig 7, we used Tg(hspGFF62A:Gal4);Tg(UAS:mem-Scarlet). All larvae were anaesthetized with 600 µM tricaine in E3 medium and immobilized in 1.5% low melting-point agarose on a coverslip glass dish and covered with tricaine/E3 medium to keep fish anaesthetized. Tiled z-stacks (with optimal z-step) were obtained using a Zeiss LSM880 microscope with Airyscan FAST in super-resolution mode, using a 20× or 40 × objective lens (Zeiss Plan-Apochromat 20 × dry, NA = 1.0; Zeiss Plan-Apochromat 40 × water-dipping, NA = 1.0). All Mauthner images (1-tile) and all lateral line images (2–4 tiles, 5% overlap) were processed using the default Airyscan processing settings (Zen Black 2.3, Zeiss). Mauthner images were taken from a lateral view of the spinal cord centered around somite 15–16 or from a dorsal view of the hindbrain. All lateral line images were taken from a lateral view of the spinal cord centered around somite 25–27. All lateral view images depict the anterior on the left and dorsal of the top, while dorsal view images depict the anterior on the top. Figure panels were prepared using Fiji (v1.51n) and Adobe Illustrator 2020 (24.0.2).

### Manual and script-based quantifications of axon diameter, Mauthner cell body and dendrite morphology

#### Manual Quantification of Mauthner diameter for VAST-SDCM images.

To quantify Mauthner axon diameter manually, z-stacks were loaded into Fiji/ImageJ (v1.51n). The look-up table (LUT) was set to “Thermal”, and images were thresholded using Min/Max. Only the top Mauthner axon (closest to the objective) was analyzed to ensure consistency with the automated image analysis pipeline. A random grid with 500 µm^2^ spacing was overlaid and every second square intersecting the top axon along its longitudinal axis was selected for measurement. Within each selected square, the Mauthner axon outline (identified by thermal LUT-highlighted turquoise pixels) was manually traced using polygon selection. A minimum of five regions distributed along the axon’s length were traced per sample. To calculate diameter, the area was divided by the corresponding width for each individual segment and the mean values across those 5–6 segments was used for analysis. A mean from those 5–6 diameter measurement was taken as the value. Because the manual measurements and the machine learning-based pipeline were trained and performed by different individuals, slight differences in axon outline definitions were expected (e.g., manual tracing based on thermal LUT-highlighted turquoise pixels versus training-based segmentation in the machine learning model). To account for this variability, axon diameter measurements were normalized by calculating the percentage change relative to the clutch-specific controls. Normalized diameter changes were then plotted as percentage differences relative to control. Only the one axon closest to the objective was quantified.

#### Script-based ImageJ Quantification of Mauthner diameter for AiryScan FAST 880 confocal images.

Axon diameter from AiryScan FAST confocal images were measured using custom-written ImageJ Macros and Fiji as described in detail in Bin and colleagues [20]. Briefly, a “Split Axons Tool” was used to split the two adjacent Mauthner axons in whole larvae z-stack datasets, resulting in two separate maximum intensity projections for each Mauthner axon. Next, the “Axon Trace Tool” was used to trace the approximate mid-point of the axon along its length according to its intensity profile. Afterwards, the “Axon Calibre Tool” was used to measure the average axon diameter along the length of the selected Mauthner axon. The diameter was measured only for the axon located closest to the imaging objective. To account for clutch-specific variability, we normalized axon diameter by calculating the change relative to DMSO-treated controls of the same clutch. Only the one axon closest to the objective was quantified.

#### Manual quantification for PLLn axon diameter for AiryScan FAST 880 confocal images.

To quantify the diameter of lateral line axons manually, z-stacks were loaded into Fiji/ImageJ (v1.51n). Single axons were outlined and manually traced using polygon selection. Only clearly distinguishable regions and myelinated axons were included in the dataset. Each animal had between 1 and 3 lateral line axons labeled. A minimum of at least 100 µm in total length was measured for each axon. To calculate diameter, the area was divided by the corresponding width for each individually traced axonal segment and the mean from the diameter of all segments was used for analysis. To account for clutch-specific variability DMSO-treated controls were always of the same clutch as drug-treated larvae and each experiment consists of data from several clutch.

#### Script-based quantification of Mauthner neuron cell body and manual quantification of dendrite and AIS size.

Seven sample images spanning the range of intensities and acquisition parameters were uploaded to ArivisCloud (Zeiss) Deep Learning platform as a training dataset. The dataset was annotated by drawing ROI examples of ‘cellBody’ and ‘background’ to multiple z-slices in each image. The model was trained and the results checked. Three iterations of annotation, training, and improvement were required to satisfactorily segment cell bodies in the sample training set. The model was imported to ArivisPro and used in a pipeline to detect cell bodies across all samples. The detections were manually quality-controlled in a blinded manner (quantified in Fig 7). To assess dendrite and AIS morphology, a maximum intensity projection of the same z-stack of the hindbrain was generated. The lateral dendrites were manually traced in Fiji, and length and area measurements were obtained. For the same images, we also manually traced the AIS and calculated the diameter by dividing the area by the corresponding width.

### Statistical analyses

Statistical analyses were performed using GraphPadPrism 10, as well as a combination of Python-based scripts and Excel-based module scripting to accommodate the large volume and complexity of screening data. *p*-values are included in the figure legends and values for z-Score ranking and p-values for the hit compounds are included in S1 Table. Significance was defined as *p* < 0.05. Unless stated otherwise, *n* number represents values from independent fish and is included in the figure legends or S1 Table. All raw values and statistical assessments can be found in the S1 Data. Power analysis and visualization was done using Anthropic. (2026). Claude Sonnet 4.6 [Large language model]. https://claude.ai/. Effect sizes were calculated as Cohen’s *d* using the pooled standard deviation of each comparison. Target power was set at 90%. All inputs including group sizes, means, standard deviations, effect sizes, and resulting power estimates are reported in the figure legends and S1 Data.

## Supporting information

S1 Video The video illustrates the stepwise object detection and processing of the Mauthner axon in Arivis Vision4D, as described in Fig 2. (MP4)

S1 Table List of hit compounds that change axon diameter. Complete list of hit compounds, putative targets and description, that change Mauthner axon diameter ranked by z-score. The z-score was calculated comparing the diameter for each compound with values of respective DMSO-treated clutch-specific controls. Student *t* test was used to additionally assess statistical significance between both groups (indicated by *). Compounds that change overall body dimensions or overall health are excluded. Class, action and description are shown for each identified hit compound.
(XLSX)

S1 Data The Source data file contains all underlying data and statistical assessment for each qualitative panel. (XLSX)

## Abbreviations

AI: artificial intelligence
AIS: axon initial segment
dpf: days post-fertilization
hpf: hours post-fertilization
ML: machine learning
PLLn: posterior lateral line nerve
SDCM: spinning disk confocal microscope
VAST: vertebrate automated screening technology
