# Supplementary material for: A high-resolution phenotypic screen identifies regulators of CNS axon diameter in zebrafish
Source: PLoS Biol. 2026 Jul 17;24(7):e3003895. doi: 10.1371/journal.pbio.3003895 (PMC13379002; doi:10.1371/journal.pbio.3003895)
Supplement: Attachment — Submitted filename: Eichel-Vogel et al_Response-to-reviewers.docx [file pbio.3003895.s006.docx]

We thank the reviewers for their thoughtful and incisive comments on our manuscript.

We have addressed their comments as detailed point by point below, and have made significant changes, adding new data and clarifying text throughout.

Of note, we have now assessed how a positive control manipulation and our validated hit compounds affect the axon diameter of another neuronal subtype (those of the peripheral lateral line nerve) that has a generally much smaller axon diameter than the Mauthner axon. We find that subtle changes in the diameter of these much smaller axons can be identified through live imaging, as assessed using the positive control manipulation, although these changes were not affected by the specific hit compounds tested. These data are now presented in an updated Figure 6.

In addition, we have assessed how our hit compounds affect other features of the Mauthner neuron, specifically cell body size, dendrite morphology and the AIS, documenting interesting differences in how the two classes of hit compounds affect neuronal morphology. These data are presented in a new figure 7.

We have also extensively rewritten our discussion in response to reviewer comments.

We hope that the reviewers now consider our manuscript suitable for publication and look forward to hearing any further feedback.

*Reviewer #1:*

*The manuscript by Eichel-Vogel et al. describes a high-throughput chemical screen in zebrafish aimed at identifying compounds that regulate axonal diameter. The authors focused on Mauthner axons because they can be labeled via transgene and are relatively large, increasing in diameter from 1 to 5 µm between 2 and 5 days post-fertilization (dpf). The study utilized a commercially available system to screen the LOPAC library, which consists of ~1,000 well-characterized compounds, many of which modulate neuronal function. To process this large dataset, the authors created a custom image analysis pipeline for quantification. Following the screen and subsequent quality control, they identified 33 compounds that either decreased or increased Mauthner axon diameter. From the primary screen, the authors selected compounds for validation based on several criteria: a substantial difference from the control, clustering within the same chemical class, cases where agonists and antagonists for the same pathway had opposite effects, and the ability to increase diameter. Based on these criteria, they validated compounds affecting beta-2-adrenergic receptors and the dopamine pathway. These validation experiments largely confirmed the findings from the original screen. The authors speculate that these compounds modulate neuronal activity, ultimately leading to changes in Mauthner axon diameter. Overall, I found the study to be well-executed, with appropriate controls and a careful evaluation of positive hits. I particularly appreciate the candid discussion regarding false positive and false negative hits in the screen. The authors have generated a valuable resource for the neuroscience community that can be mined for pathways regulating axon diameter.
However, the study does have several shortcomings. First, it is not immediately clear whether this approach can be translated to other, smaller axons. It would be useful to know whether the identified compounds affect the diameter of other axon types. Finally, the study is not particularly mechanistic. While the authors speculate on how the modulation of beta-2-adrenergic and dopamine pathways may affect diameter, they do not experimentally test this theory.*

We thank the reviewer for their generally positive assessment and hope to have addressed their comments sufficiently, as noted below.

*Specific Comments:*

*1. Resolution Limits: The authors need to discuss the limitations of the VAST screening system more explicitly. They utilized a 20x objective, yielding a resolution of 0.286 µm/pixel. Based on the Nyquist sampling theorem (which ideally requires sampling >2 times per resolution unit), this resolution may not be sufficient to detect changes in smaller axons (≤ 1 µm). It is my understanding that most axons in zebrafish are less than 1 µm in diameter. While it is reasonable to assume that many pathways identified in the Mauthner axon screen are conserved and could be examined in other systems, this technical limitation regarding generalizability needs to be mentioned.*

We agree entirely with the reviewer and have extensively revised our discussion. We now specifically address the issue of how the resolution of image acquisition affects the ability to detect changes in axon diameter. We speak to how the resolution limit of the system that we employed for our reported screen was such that assessing changes to Mauthner axon was the most feasible and look ahead to what innovations in image acquisition might be required to carry out discovery screens that aim to assess changes to the diameter of smaller axons.

It is important to note that we now include assessment of the ability to detect changes to the diameter of much smaller posterior lateral line nerve axons, showing that it is possible to do so through a live imaging approach, but using a different imaging platform (updated Figure 6). We discuss how working towards automated image acquisition on higher resolution systems may unlock our ability to screen for changes to further fine properties of neuronal morphology.

*2. Broad Applicability: The study would greatly benefit from demonstrating that at least one or more of the identified compounds functions broadly in the nervous system (i.e., that their findings are not restricted solely to Mauthner axons).*

We have now included an assessment of how a positive control manipulation and our hit compounds affect the diameter of axons of the posterior lateral line nerve. These axons are of a considerably smaller than the Mauthner axon, being ~1µm in diameter. Nonetheless, through live imaging fluorescent reporters expressed by these neurons, we were able to detect subtle changes to their diameter. As it happens, the validated hits did not significantly affect the diameter of these axons, although one compound did demonstrate a trend towards increased diameter in line with the direction of effect on the Mauthner axon (see updated Figure 6).

*3. Mechanism of Action: Can the authors demonstrate that compounds modulating beta-2-adrenergic receptors and dopamine pathways actually change Mauthner cell activity? Confirming this would provide a mechanistic underpinning for the observed phenotype.*

We thank the reviewer for this comment and realise that we had perhaps overstated a potential relationship between neuronal activity and axon diameter in our original discussion as it related to the growth of the Mauthner axon. The Mauthner axon is a command neuron that is only activated upon detection of aversive sensory stimuli, e.g. presence of a predator, and so is largely inactive during development. It is, however, possible that the compound treatments could make the Mauthner neuron more excitable to otherwise sub-threshold stimuli. To assess this, we developed a sensory stimulation paradigm calibrated to induce responses of the Mauthner neuron (assessed by Ca2+ imaging) at a variable rate. Using this we found no difference in the response rate of the Mauthner axon between hit treatment compared and controls. We have chosen not to include these data, as we feel that the set-up logic for this experiment may be confusing to readers, given the general inactivity of Mauthner in the zebrafish. Therefore, we have toned down discussion about the role of neuronal activity in regulating Mauthner axon diameter.

Instead, we now include a broader assessment of how our hits affect other features of the morphology of the Mauthner neuron. We find that different compounds have distinct effects, with the dopamine antagonists decreasing cell body size while increasing axon diameter, but the beta2 adrenoceptor agonists generally influencing cell size (both cell body and axon diameter). We agree that detailed mechanistic interrogation will be required to disentangle how these compounds and their targets in vivo affect neuronal morphology including axon diameter.

*Reviewer #2: In this manuscript by Eichel-Vogel et al the authors report on a pipeline for a high resolution medium throughput screen of compounds that affect axon diameter using zebrafish. Overall, the authors provide a good rationale for developing and validating their pipeline. Using the pipeline, they perform a well-controlled small molecule screen that yields quantifiable  results comparing the effects of over 800 compounds on axon diameter. The authors identify and validate several novel regulators for which further characterization in the future seems essential to determine their impact. As with most screening platform, there remain a handful of caveats that the authors acknowledge. Overall, this manuscript seems adequate for the purview of PLOS Biology Methods and Resources and will be of interest to a broad readership in the field of neuroscience, zebrafish biology, developmental biology, among others.*

We thank the reviewer for their positive assessment and support, and hope to have addressed their key points below.

*Major Points:*

*1. 17.5% technical exclusion is rather high. It would be helpful for the authors to comment further on this and provide more detail as to why this was the case and how it could be improved upon going forward.*

We thank the reviewer for making this point and it is entirely correct that this is a relatively high technical exclusion rate. We have greatly extended our discussion to explain the basis of this exclusion rate, which is largely driven by the challenges of a screen that aimed to detect subtle changes to cell morphology.

Technical exclusions were largely driven by the stringent need for high-resolution imaging that allowed accurate assessment of Mauthner axon diameter. If animals drifted at all during image acquisition (noting that they reside in a glass capillary while imaging) this could lead to blurring that resulted in their exclusion. Similarly, if there were any artifacts caused by additional factors during imaging, e.g. presence of ectopic cells expressing a reporter elsewhere in the z-stack, this may have resulted in exclusion. In our revised discussion, we also give considerations as to how future screens aimed at identifying alterations at this scale of resolution might be improved. For example, new technologies allow facile targeted knock in of fluorescent reporters into endogenous loci that typically exhibit much more consistent expression of reporters animal to animal.

*2. The authors identify two pathways from their chemical screen that largely phenocopy in their follow up experiments. However, one chemical does not (Fenspiride hydrochloride). It would be helpful to know if they returned to the original data and analyzed it manually to see if it was a true false positive or if the algorithm miscalculated the diameter change.*

The reviewer brings up the very important consideration of comparing machine-learning based measurements with manual measurements. As prompted by the reviewer we returned to the original dataset from the screen and carried out an analysis that compared the original machine-learning based measurements with a manual analysis (shown below). It is important to note that this compound was deemed a hit by our z-score ranking in the original screen, but not when subjected to our subsequent hit to control statistical comparison. Our subsequent manual reanalysis of this compound substantiates this conclusion, pointing to a trend decrease in axon diameter. It is important to note that the ability to identify a subtle effect of this compound was not sufficiently well powered in our screen, as only 5 animals passed QC for analysis in our screen. Therefore, our conclusion is that this compound reflects a false positive.

Based on the reviewers comment we also decided to formally compare measurements made by the machine learning pipeline and two different manual assessors. Strikingly we found that while the machine learning pipeline returned values different to the manual assessors, the measured values made by the manual assessors also significantly differed from one another, highlighting the importance of an objective automated process for image analysis (see updated Figure 3).

*3. Given the rather small effect sizes observed following drug treatment, it would be extremely helpful to know what the natural variation of M cell axon diameter is across the most commonly used laboratory zebrafish strains.*

The issue of variation in Mauthner axon diameter is an important consideration. Indeed, our work over the years has indicated that axon diameter can be very sensitive to environmental conditions as animals grow and so one must be very rigorous in maintaining rigid environmental control when planning to assess axon diameter. We haven’t compared axon diameter between strains as part of the revision, but we have added discussion to the manuscript to indicate how the diameter can even vary significantly between animals of the same clutch. This has a direct influence on the ability to detect meaningful and real changes in diameter, and we discuss this constraint/ consideration in detail now, so that the community is aware. Furthermore, we include data related an important point that we had noted in passing in our original submission, namely the effect of the vehicle DMSO on axon diameter. We now show in an updated Figure 1 that while 0.5% DMSO does not affect axon diameter compared to fish water alone, just 1% DMSO can indeed influence axon diameter, highlighting how much care must be given to growth conditions when assessing this feature of neuronal morphology. We also note in our updated discussion how our efforts to identify specific regulators of axon diameter may have influenced the effect size that was possible to achieve: we applied drugs only from 3-5 days post-fertilisation, a period after axonal outgrowth and during axon diameter growth. However, although the axons do grow over this time, they only grow about 1.5µm in diameter. Therefore, this restricts the absolute changes that are likely to be driven by specific regulation of axon diameter growth, as opposed to general toxic effects, for example. Although our effect sizes may appear relatively small, as presented with respect to the absolute size of the axon, they should be taken in the context of the time over which the compounds could have affected axon diameter growth.

*4. For the two drugs that increase axonal M cell diameter, is this due to a more general mechanism active in all neurons or is it selective to this neuronal cell type. Measuring the effects of the two drugs on e.g. lateral line axons also labeled by this promotor would provide important information.*

We thank the reviewer for this important question. As they note, the reporter line that we used in our screen also labels a subset of lateral line axons. We have now assessed how our compounds affect the diameter of these axons, finding that our beta2 adrenoceptor agonists lead to a trend increase in diameter, but the D2 antagonists have no effect. Importantly, we used a positive control manipulation to demonstrate that our live imaging approach can detect changes to the diameter of these lateral line axons. These new data are shown in updated Figure 6.

*5. Similarly, do the two drugs alter other aspects of M cell morphology such as dendrite morphology? This should be easy to determine and would provide information regarding the specificity of the drugs/signaling pathways on axon diameter.*

We thank the reviewer for this question as well and we have now assessed how our hit compounds affect other features of Mauthner cell morphology. Interestingly, while the beta2 adrenoceptor agonists seemed to drive a general increase in cell growth, the D2 receptor antagonists led to a surprising phenotype, wherein cell body size was actually decreased alongside the increase to axon diameter. These data are all shown in a new figure 7. Going forward it will certainly be informative to compare how changes to axon diameter relate to alterations of other features of neuronal morphology, a point we emphasise further in our updated discussion.

*This technology relies heavily on the image segmentation and classification to enable high throughput analysis. It is unclear how these scripts will be distributed to the community. Inclusion of ImageJ plugin information or a github repository to access the code would be helpful for making this resource widely available.*

We absolutely agree and we have now deposited all scripts and pipelines at on our local Edinburgh Datashare servers, and we will make these openly accessible through a dedicated link, already noted in our manuscript, upon acceptance.

*Minor Points:*

*1. For the uninitiated reader,  it will be helpful to elaborate in the introduction on the current knowledge regarding what factors and mechanism contribute to axon diameter (neurofilaments, cytoskeleton, etc.).*  

Thank you. We have updated our introduction accordingly.

*The method is dependent upon a specific proprietary platform (VAST) limiting its wide adoption.*

It is true that our screen employed a proprietary system. In our expanded discussion we do indicate the emergence of alternative technologies and of other approaches that may be employed to execute ever-more refined screens to assess features of neuronal morphology.

*2. Figure 3 validates the pipeline with comparison of automated and manual quantification. It would be helpful to see the direct comparisons in B and C. Such that quantified area of the same axon by both measures shown visually in B and lines connecting corresponding dots in C shown.*

We have updated Figure 3 to address this point. We have additionally included a direct comparison of the automated analysis and two different manual assessors, which indicate significant difference in measurements made by manual assessors, highlighting the importance of unbiased automated analyses.

*3. Figure 4F uses Student's t-test, whereas ANOVA with multiple comparisons test would be more appropriate for the dataset*

It is true that ANOVA is the more appropriate test for multiple comparisons, but we clarify in the revised manuscript that we triaged potential hits through two steps. In the first case we simply rank compounds through z-score assessment as is common in large screens with complex quantitative readouts. We then subsequently compare individual compounds with their respective plate controls using the Student’s t-test as a second triage step, rather than as a formal statistical assessment per se. The most important tests for validating compound activities after the screen indeed use ANOVA as the statistical approach.
