# Supplementary material for: A high-resolution phenotypic screen identifies regulators of CNS axon diameter in zebrafish
Source: PLoS Biol. 2026 Jul 17;24(7):e3003895. doi: 10.1371/journal.pbio.3003895 (PMC13379002; doi:10.1371/journal.pbio.3003895)
Supplement: Peer review history

# Editor report: Decision Letter 0

**DOI:** 10.1371/journal.pbio.3003895.r001

**Authors:** Taylor Hart, PhD

12 Dec 2025

Dear Dr Lyons,

Thank you for submitting your manuscript entitled "High-resolution phenotypic screen in zebrafish for novel regulators of CNS axon diameter growth" for consideration as a Methods and Resources by PLOS Biology.

Your manuscript has now been evaluated by the PLOS Biology editorial staff, as well as by an academic editor with relevant expertise, and I am writing to let you know that we would like to send your submission out for external peer review.

However, before we can send your manuscript to reviewers, we need you to complete your submission by providing the metadata that is required for full assessment. To this end, please login to Editorial Manager where you will find the paper in the 'Submissions Needing Revisions' folder on your homepage. Please click 'Revise Submission' from the Action Links and complete all additional questions in the submission questionnaire.

Once your full submission is complete, your paper will undergo a series of checks in preparation for peer review. After your manuscript has passed the checks it will be sent out for review. To provide the metadata for your submission, please Login to Editorial Manager (https://www.editorialmanager.com/pbiology) within two working days, i.e. by Dec 14 2025 11:59PM.

If your manuscript has been previously peer-reviewed at another journal, PLOS Biology is willing to work with those reviews in order to avoid re-starting the process. Submission of the previous reviews is entirely optional and our ability to use them effectively will depend on the willingness of the previous journal to confirm the content of the reports and share the reviewer identities. Please note that we reserve the right to invite additional reviewers if we consider that additional/independent reviewers are needed, although we aim to avoid this as far as possible. In our experience, working with previous reviews does save time.

If you would like us to consider previous reviewer reports, please edit your cover letter to let us know and include the name of the journal where the work was previously considered and the manuscript ID it was given. In addition, please upload a response to the reviews as a 'Prior Peer Review' file type, which should include the reports in full and a point-by-point reply detailing how you have or plan to address the reviewers' concerns.

During the process of completing your manuscript submission, you will be invited to opt-in to posting your pre-review manuscript as a bioRxiv preprint. Visit http://journals.plos.org/plosbiology/s/preprints for full details. If you consent to posting your current manuscript as a preprint, please upload a single Preprint PDF.

Feel free to email us at plosbiology@plos.org if you have any queries relating to your submission.

Kind regards,

Taylor

Taylor Hart, PhD,

Associate Editor

PLOS Biology

thart@plos.org

# Peer review: Decision Letter 1

**DOI:** 10.1371/journal.pbio.3003895.r002

**Authors:** Taylor Hart, PhD, Taylor Hart, PhD

28 Jan 2026

Dear Dr Lyons,

Thank you for your patience while your manuscript "High-resolution phenotypic screen in zebrafish for novel regulators of CNS axon diameter growth" was peer-reviewed at PLOS Biology. It has now been evaluated by the PLOS Biology editors, an Academic Editor with relevant expertise, and by several independent reviewers.

In light of the reviews, which you will find at the end of this email, we would like to invite you to revise the work to thoroughly address the reviewers' reports.

The reviewers wrote that the study appears well-executed and that the results will be of interest. However, they both brought up areas where the broad applicability of the results could be strengthened by examining other neuron types or properties. They also commented on technical aspects and areas requiring clarification. Reviewer 2 raised a statistical concern. In your revision, you should examine effects on other axon types as outlined by Reviewer 2 in Major Point 4, and thoroughly address all other points raised by the reviewers. It is beyond the scope of this Methods & Resources submission to require mechanistic investigations, but you should discuss these points in a new paragraph defining the limitations of the study. Per Reviewer 2's comment about code availability, PLOS Biology requires that you make available all custom code and scripts used in the study.

Given the extent of revision needed, we cannot make a decision about publication until we have seen the revised manuscript and your response to the reviewers' comments. Your revised manuscript is likely to be sent for further evaluation by all or a subset of the reviewers.

In addition to these revisions, you will need to complete some formatting changes, which you will receive in a follow up email. A member of our team will be in touch with a set of requests shortly.

We expect to receive your revised manuscript within 3 months. Please email us (plosbiology@plos.org) if you have any questions or concerns, or would like to request an extension.

At this stage, your manuscript remains formally under active consideration at our journal; please notify us by email if you do not intend to submit a revision so that we may withdraw it.

**IMPORTANT - SUBMITTING YOUR REVISION**

Your revisions should address the specific points made by each reviewer. Please submit the following files along with your revised manuscript:

1. A 'Response to Reviewers' file - this should detail your responses to the editorial requests, present a point-by-point response to all of the reviewers' comments, and indicate the changes made to the manuscript.

*NOTE: In your point-by-point response to the reviewers, please provide the full context of each review. Do not selectively quote paragraphs or sentences to reply to. The entire set of reviewer comments should be present in full and each specific point should be responded to individually, point by point.

You should also cite any additional relevant literature that has been published since the original submission and mention any additional citations in your response.

2. In addition to a clean copy of the manuscript, please also upload a 'track-changes' version of your manuscript that specifies the edits made. This should be uploaded as a "Revised Article with Changes Highlighted" file type.

*Re-submission Checklist*

When you are ready to resubmit your revised manuscript, please refer to this re-submission checklist: https://plos.io/Biology_Checklist

To submit a revised version of your manuscript, please go to https://www.editorialmanager.com/pbiology/ and log in as an Author. Click the link labelled 'Submissions Needing Revision' where you will find your submission record.

Please make sure to read the following important policies and guidelines while preparing your revision:

*Published Peer Review*

Please note while forming your response, if your article is accepted, you may have the opportunity to make the peer review history publicly available. The record will include editor decision letters (with reviews) and your responses to reviewer comments. If eligible, we will contact you to opt in or out. Please see here for more details:

https://blogs.plos.org/plos/2019/05/plos-journals-now-open-for-published-peer-review/

*PLOS Data Policy*

Please note that as a condition of publication PLOS' data policy (http://journals.plos.org/plosbiology/s/data-availability) requires that you make available all data used to draw the conclusions arrived at in your manuscript. If you have not already done so, you must include any data used in your manuscript either in appropriate repositories, within the body of the manuscript, or as supporting information (N.B. this includes any numerical values that were used to generate graphs, histograms etc.). For an example see here: http://www.plosbiology.org/article/info%3Adoi%2F10.1371%2Fjournal.pbio.1001908#s5

*Blot and Gel Data Policy*

We require the original, uncropped and minimally adjusted images supporting all blot and gel results reported in an article's figures or Supporting Information files. We will require these files before a manuscript can be accepted so please prepare them now, if you have not already uploaded them. Please carefully read our guidelines for how to prepare and upload this data: https://journals.plos.org/plosbiology/s/figures#loc-blot-and-gel-reporting-requirements

*Protocols deposition*

To enhance the reproducibility of your results, we recommend that if applicable you deposit your laboratory protocols in protocols.io, where a protocol can be assigned its own identifier (DOI) such that it can be cited independently in the future. Additionally, PLOS ONE offers an option for publishing peer-reviewed Lab Protocol articles, which describe protocols hosted on protocols.io. Read more information on sharing protocols at https://plos.org/protocols?utm_medium=editorial-email&utm_source=authorletters&utm_campaign=protocols

Thank you again for your submission to our journal. We hope that our editorial process has been constructive thus far, and we welcome your feedback at any time. Please don't hesitate to contact us if you have any questions or comments.

Sincerely,

Taylor

Taylor Hart, PhD,

Associate Editor

PLOS Biology

thart@plos.org

------------------------------------

REVIEWS:

Reviewer #1: The manuscript by Eichel-Vogel et al. describes a high-throughput chemical screen in zebrafish aimed at identifying compounds that regulate axonal diameter. The authors focused on Mauthner axons because they can be labeled via transgene and are relatively large, increasing in diameter from 1 to 5 µm between 2 and 5 days post-fertilization (dpf).

The study utilized a commercially available system to screen the LOPAC library, which consists of ~1,000 well-characterized compounds, many of which modulate neuronal function. To process this large dataset, the authors created a custom image analysis pipeline for quantification. Following the screen and subsequent quality control, they identified 33 compounds that either decreased or increased Mauthner axon diameter.

From the primary screen, the authors selected compounds for validation based on several criteria: a substantial difference from the control, clustering within the same chemical class, cases where agonists and antagonists for the same pathway had opposite effects, and the ability to increase diameter. Based on these criteria, they validated compounds affecting beta-2-adrenergic receptors and the dopamine pathway. These validation experiments largely confirmed the findings from the original screen. The authors speculate that these compounds modulate neuronal activity, ultimately leading to changes in Mauthner axon diameter.

Overall, I found the study to be well-executed, with appropriate controls and a careful evaluation of positive hits. I particularly appreciate the candid discussion regarding false positive and false negative hits in the screen. The authors have generated a valuable resource for the neuroscience community that can be mined for pathways regulating axon diameter.

However, the study does have several shortcomings. First, it is not immediately clear whether this approach can be translated to other, smaller axons. It would be useful to know whether the identified compounds affect the diameter of other axon types. Finally, the study is not particularly mechanistic. While the authors speculate on how the modulation of beta-2-adrenergic and dopamine pathways may affect diameter, they do not experimentally test this theory.

Specific Comments:

1.Resolution Limits: The authors need to discuss the limitations of the VAST screening system more explicitly. They utilized a 20x objective, yielding a resolution of 0.286 µm/pixel. Based on the Nyquist sampling theorem (which ideally requires sampling >2 times per resolution unit), this resolution may not be sufficient to detect changes in smaller axons (≤ 1 µm). It is my understanding that most axons in zebrafish are less than 1 µm in diameter. While it is reasonable to assume that many pathways identified in the Mauthner axon screen are conserved and could be examined in other systems, this technical limitation regarding generalizability needs to be mentioned.

2.Broad Applicability: The study would greatly benefit from demonstrating that at least one or more of the identified compounds functions broadly in the nervous system (i.e., that their findings are not restricted solely to Mauthner axons).

3.Mechanism of Action: Can the authors demonstrate that compounds modulating beta-2-adrenergic receptors and dopamine pathways actually change Mauthner cell activity? Confirming this would provide a mechanistic underpinning for the observed phenotype.

Reviewer #2: In this manuscript by Eichel-Vogel et al the authors report on a pipeline for a high resolution medium throughput screen of compounds that affect axon diameter using zebrafish. Overall, the authors provide a good rationale for developing and validating their pipeline. Using the pipeline they perform a well controlled small molecule screen that yields quantifiable results comparing the effects of over 800 compounds on axon diameter. The authors identify and validate several novel regulators for which further characterization in the future seems essential to determine their impact. As with most screening platform, there remain a handful of caveats that the authors acknowledge. Overall, this manuscript seems adequate for the purview of PLOS Biology Methods and Resources and will be of interest to a broad readership in the field of neuroscience, zebrafish biology, developmental biology, among others.

Major Points:

1. 17.5% technical exclusion is rather high. It would be helpful for the authors to comment further on this and provide more detail as to why this was the case and how it could be improved upon going forward.

2. The authors identify two pathways from their chemical screen that largely phenocopy in their follow up experiments. However, one chemical does not (Fenspiride hydrochloride). It would be helpful to know if they returned to the original data and analyzed it manually to see if it was a true false positive or if the algorithm miscalculated the diameter change.

3. Given the rather small effect sizes observed following drug treatment, it would be extremely helpful to know what the natural variation of M cell axon diameter is across the most commonly used laboratory zebrafish strains.

4. For the two drugs that increase axonal M cell diameter, is this due to a more general mechanism active in all neurons or is it selective to this neuronal cell type. Measuring the effects of the two drugs on e.g. lateral line axons also labeled by this promotor would provide important information.

5. Similarly, do the two drugs alter other aspects of M cell morphology such as dendrite morphology? This should be easy to determine and would provide information regarding the specificity of the drugs/signaling pathways on axon diameter.

This technology relies heavily on the image segmentation and classification to enable high throughput analysis. It is unclear how these scripts will be distributed to the community. Inclusion of ImageJ plugin information or a github repository to access the code would be helpful for making this resource widely available.

Minor Points:

1. For the uninitiated reader, it will be helpful to elaborate in the introduction on the current knowledge regarding what factors and mechanism contribute to axon diameter (neurofilaments, cytoskeleton, etc.).

The method is dependent upon a specific proprietary platform (VAST) limiting its wide adoption.

2. Figure 3 validates the pipeline with comparison of automated and manual quantification. It would be helpful to see the direct comparisons in B and C. Such that quantified area of the same axon by both measures shown visually in B and lines connecting corresponding dots in C shown.

3. Figure 4F uses Student's t-test, whereas ANOVA with multiple comparisons test would be more appropriate for the dataset.

# Author comment: Author response to Decision Letter 2

**DOI:** 10.1371/journal.pbio.3003895.r003

22 May 2026

Attachment Submitted filename: Eichel-Vogel et al_Response-to-reviewers.docx

# Editor report: Decision Letter 2

**DOI:** 10.1371/journal.pbio.3003895.r004

**Authors:** Taylor Hart, PhD, Taylor Hart, PhD, Taylor Hart, PhD

4 Jun 2026

Dear Dr Lyons,

Thank you for your patience while we considered your revised manuscript "High-resolution phenotypic screen in zebrafish for novel regulators of CNS axon diameter growth" for publication as a Methods and Resources article at PLOS Biology. This revised version of your manuscript has been evaluated by the PLOS Biology editors and the Academic Editor.

Based on our Academic Editor's assessment of your revision, we are likely to accept this manuscript for publication, provided you satisfactorily address some additional minor points raised by the Academic Editor (see below my signature). Please also make sure to address the following data and other policy-related requests.

IMPORTANT: Please ensure that your next revision also addresses the following points:

------------------------

**Title:

We would like to tweak the title to better align with our stylistic preferences. Is the following alternative version acceptable to you?

"A high-resolution phenotypic screen identifies regulators of CNS axon diameter in zebrafish"

**Data and Supplement:

Thank you for providing the underlying data in the supplement. We request a few minor changes to this.

First, we didn't see underlying data labeled as "6J", but this may be due to a labeling error in the excel file. Please double check this and update if appropriate.

Second, please change the file names of the supplementary items to match our formatting requirements, i.e. "S1_Table.xlsx" and "S2_Data.xlsx".

Third, please cite the location of the underlying data in all relevant figure legends, e.g. "The data underlying this figure can be found in S2 Data."

**Code availability:

Per journal policy, if you have generated any custom code or scripts during the course of this investigation, we require that you make it available without restrictions. Please ensure that the code is sufficiently well documented and reusable, and that your Data Statement in the Editorial Manager submission system accurately describes where your code can be found. Please also ensure that you choose a license for your code and include a Readme file.

Please note that we cannot accept sole deposition of code in GitHub, as this could be changed after publication. However, you can archive this version of your publicly available GitHub code to Zenodo. Once you do this, it will generate a DOI number, which you will need to provide in the Data Accessibility Statement (you are welcome to also provide the GitHub access information). See the process for doing this here: https://docs.github.com/en/repositories/archiving-a-github-repository/referencing-and-citing-content

-----------------------

As you address these items, please take this last chance to review your reference list to ensure that it is complete and correct. If you have cited papers that have been retracted, please include the rationale for doing so in the manuscript text, or remove these references and replace them with relevant current references. Any changes to the reference list should be mentioned in the cover letter that accompanies your revised manuscript.

In addition to these revisions, you may need to complete some formatting changes, which you will receive in a follow up email. A member of our team will be in touch with a set of requests shortly. If you do not receive a separate email within a few days, please assume that checks have been completed, and no additional changes are required.

We expect to receive your revised manuscript within two weeks.

To submit your revision, please go to https://www.editorialmanager.com/pbiology/ and log in as an Author. Click the link labelled 'Submissions Needing Revision' to find your submission record. Your revised submission must include the following:

- a cover letter that should detail your responses to any editorial requests, if applicable, and whether changes have been made to the reference list

- a Response to Reviewers file that provides a detailed response to the reviewers' comments (if applicable, if not applicable please do not delete your existing 'Response to Reviewers' file.)

- a track-changes file indicating any changes that you have made to the manuscript.

NOTE: If Supporting Information files are included with your article, note that these are not copyedited and will be published as they are submitted. Please ensure that these files are legible and of high quality (at least 300 dpi) in an easily accessible file format. For this reason, please be aware that any references listed in an SI file will not be indexed. For more information, see our Supporting Information guidelines:

https://journals.plos.org/plosbiology/s/supporting-information

*Published Peer Review History*

Please note that you may have the opportunity to make the peer review history publicly available. The record will include editor decision letters (with reviews) and your responses to reviewer comments. If eligible, we will contact you to opt in or out. Please see here for more details:

https://plos.org/published-peer-review-history/

*Press*

Should you, your institution's press office or the journal office choose to press release your paper, please ensure you have opted out of Early Article Posting on the submission form. We ask that you notify us as soon as possible if you or your institution is planning to press release the article.

*Protocols deposition*

To enhance the reproducibility of your results, we recommend that if applicable you deposit your laboratory protocols in protocols.io, where a protocol can be assigned its own identifier (DOI) such that it can be cited independently in the future. Additionally, PLOS ONE offers an option for publishing peer-reviewed Lab Protocol articles, which describe protocols hosted on protocols.io. Read more information on sharing protocols at https://plos.org/protocols?utm_medium=editorial-email&utm_source=authorletters&utm_campaign=protocols

Please do not hesitate to contact me should you have any questions.

Sincerely,

Taylor

Taylor Hart, PhD,

Associate Editor

thart@plos.org

PLOS Biology

Comments from the Academic Editor:

On line 81, please italicize the zebrafish gene

Please change line 477 from "n number" to "sample size" (and throughout that paragraph)

Please italicize in vivo throughout.

# Author comment: Author response to Decision Letter 3

**DOI:** 10.1371/journal.pbio.3003895.r005

17 Jun 2026

Attachment Submitted filename: Eichel-Vogel_et_al_Response-to-reviewers_auresp_3.docx

# Editor report: Decision Letter 3

**DOI:** 10.1371/journal.pbio.3003895.r006

**Authors:** Taylor Hart, PhD, Taylor Hart, PhD, Taylor Hart, PhD, Taylor Hart, PhD

23 Jun 2026

Dear Dr Lyons,

Thank you for the submission of your revised Methods and Resources "A high-resolution phenotypic screen identifies regulators of CNS axon diameter in zebrafish" for publication in PLOS Biology. On behalf of my colleagues and the Academic Editor, Cody Smith, I am pleased to say that we can in principle accept your manuscript for publication, provided you address any remaining formatting and reporting issues. These will be detailed in an email you should receive within 2-3 business days from our colleagues in the journal operations team; no action is required from you until then. Please note that we will not be able to formally accept your manuscript and schedule it for publication until you have completed any requested changes.

Please take a minute to log into Editorial Manager at http://www.editorialmanager.com/pbiology/, click the "Update My Information" link at the top of the page, and update your user information to ensure an efficient production process.

PRESS

We frequently collaborate with press offices. If your institution or institutions have a press office, please notify them about your upcoming paper at this point, to enable them to help maximise its impact. If the press office is planning to promote your findings, we would be grateful if they could coordinate with biologypress@plos.org. If you have previously opted in to the early version process, we ask that you notify us immediately of any press plans so that we may opt out on your behalf.

We also ask that you take this opportunity to read our Embargo Policy regarding the discussion, promotion and media coverage of work that is yet to be published by PLOS. As your manuscript is not yet published, it is bound by the conditions of our Embargo Policy. Please be aware that this policy is in place both to ensure that any press coverage of your article is fully substantiated and to provide a direct link between such coverage and the published work. For full details of our Embargo Policy, please visit http://www.plos.org/about/media-inquiries/embargo-policy/.

Thank you again for choosing PLOS Biology for publication and supporting Open Access publishing. We look forward to publishing your study.

Sincerely,

Taylor

Taylor Hart, PhD,

Associate Editor

PLOS Biology

thart@plos.org
